# Developmental pathways to adiposity begin before birth and are influenced by genotype, prenatal environment and epigenome

**DOI:** 10.1186/s12916-017-0800-1

**Published:** 2017-03-07

**Authors:** Xinyi Lin, Ives Yubin Lim, Yonghui Wu, Ai Ling Teh, Li Chen, Izzuddin M. Aris, Shu E. Soh, Mya Thway Tint, Julia L. MacIsaac, Alexander M. Morin, Fabian Yap, Kok Hian Tan, Seang Mei Saw, Michael S. Kobor, Michael J. Meaney, Keith M. Godfrey, Yap Seng Chong, Joanna D. Holbrook, Yung Seng Lee, Peter D. Gluckman, Neerja Karnani, on behalf of the GUSTO study group, Pratibha Agarwal, Arijit Biswas, Choon Looi Bong, Birit F.P. Broekman, Shirong Cai, Jerry Kok Yen Chan, Yiong Huak Chan, Cornelia Yin Ing Chee, Helen Chen, Yin Bun Cheung, Amutha Chinnadurai, Chai Kiat Chng, Mary Foong-Fong Chong, Yap-Seng Chong, Shang Chee Chong, Mei Chien Chua, Doris Fok, Marielle V. Fortier, Peter D. Gluckman, Keith M. Godfrey, Anne Eng Neo Goh, Yam Thiam Daniel Goh, Joshua J. Gooley, Wee Meng Han, Mark Hanson, Christiani Jeyakumar Henry, Joanna D. Holbrook, Chin-Ying Hsu, Neerja Karnani, Jeevesh Kapur, Kenneth Kwek, Ivy Yee-Man Lau, Bee Wah Lee, Yung Seng Lee, Ngee Lek, Sok Bee Lim, Iliana Magiati, Lourdes Mary Daniel, Michael Meaney, Cheryl Ngo, Krishnamoorthy Niduvaje, Wei Wei Pang, Anqi Qiu, Boon Long Quah, Victor Samuel Rajadurai, Mary Rauff, Salome A. Rebello, Jenny L. Richmond, Anne Rifkin-Graboi, Seang-Mei Saw, Lynette Pei-Chi Shek, Allan Sheppard, Borys Shuter, Leher Singh, Shu-E Soh, Walter Stunkel, Lin Lin Su, Kok Hian Tan, Oon Hoe Teoh, Mya Thway Tint, Hugo P S van Bever, Rob M. van Dam, Inez Bik Yun Wong, P. C. Wong, Fabian Yap, George Seow Heong Yeo

**Affiliations:** 1 Singapore Institute for Clinical Sciences, A*STAR, 30 Medical Drive, Singapore, 117609 Singapore; 2 Department of Obstetrics and Gynaecology, Yong Loo Lin School of Medicine, National University of Singapore, Singapore, 119228 Singapore; 3 Department of Pediatrics, Yong Loo Lin School of Medicine, National University of Singapore, Singapore, 119228 Singapore; 4 Centre for Molecular Medicine and Therapeutics, Child and Family Research Institute, Department of Medical Genetics, University of British Columbia, Vancouver, BC V5Z 4H4 Canada; 5 KK Women’s and Children’s Hospital, Singapore, 229899 Singapore; 6 Saw Swee Hock School of Public Health, National University of Singapore, Singapore, 117597 Singapore; 7 Singapore Eye Research Institute, Singapore, 169856 Singapore; 8 Duke NUS Medical School, Singapore, 169857 Singapore; 9 Ludmer Centre for Neuroinformatics and Mental Health, Douglas University Mental Health Institute, McGill University, Montreal, Quebec H4H 1R3 Canada; 10 MRC Lifecourse Epidemiology Unit and NIHR Southampton Biomedical Research Centre, University of Southampton and University Hospital Southampton NHS Foundation Trust, Southampton, SO16 6YD UK; 11 Division of Paediatric Endocrinology and Diabetes, Khoo Teck Puat-National University Children’s Medical Institute, National University Health System, Singapore, 119228 Singapore; 12 Centre for Human Evolution, Adaptation and Disease, Liggins Institute, University of Auckland, Auckland, 1142 New Zealand; 13 Department of Biochemistry, Yong Loo Lin School of Medicine, National University of Singapore, Singapore, 119228 Singapore

**Keywords:** Epigenome-wide association study, Offspring adiposity, DNA methylation, Prenatal environment, Birth weight

## Abstract

**Background:**

Obesity is an escalating health problem worldwide, and hence the causes underlying its development are of primary importance to public health. There is growing evidence that suboptimal intrauterine environment can perturb the metabolic programing of the growing fetus, thereby increasing the risk of developing obesity in later life. However, the link between early exposures in the womb, genetic susceptibility, and perturbed epigenome on metabolic health is not well understood. In this study, we shed more light on this aspect by performing a comprehensive analysis on the effects of variation in prenatal environment, neonatal methylome, and genotype on birth weight and adiposity in early childhood.

**Methods:**

In a prospective mother-offspring cohort (N = 987), we interrogated the effects of 30 variables that influence the prenatal environment, umbilical cord DNA methylation, and genotype on offspring weight and adiposity, over the period from birth to 48 months. This is an interim analysis on an ongoing cohort study.

**Results:**

Eleven of 30 prenatal environments, including maternal adiposity, smoking, blood glucose and plasma unsaturated fatty acid levels, were associated with birth weight. Polygenic risk scores derived from genetic association studies on adult adiposity were also associated with birth weight and child adiposity, indicating an overlap between the genetic pathways influencing metabolic health in early and later life. Neonatal methylation markers from seven gene loci (*ANK3*, *CDKN2B*, *CACNA1G*, *IGDCC4*, *P4HA3*, *ZNF423* and *MIRLET7BHG*) were significantly associated with birth weight, with a subset of these in genes previously implicated in metabolic pathways in humans and in animal models. Methylation levels at three of seven birth weight-linked loci showed significant association with prenatal environment, but none were affected by polygenic risk score. Six of these birth weight-linked loci continued to show a longitudinal association with offspring size and/or adiposity in early childhood.

**Conclusions:**

This study provides further evidence that developmental pathways to adiposity begin before birth and are influenced by environmental, genetic and epigenetic factors. These pathways can have a lasting effect on offspring size, adiposity and future metabolic outcomes, and offer new opportunities for risk stratification and prevention of obesity.

**Clinical Trial Registration:**

This birth cohort is a prospective observational study, designed to study the developmental origins of health and disease, and was retrospectively registered on 1 July 2010 under the identifier NCT01174875.

**Electronic supplementary material:**

The online version of this article (doi:10.1186/s12916-017-0800-1) contains supplementary material, which is available to authorized users.

## Background

The epidemic of obesity is a major public health issue. The risk of obesity appears to begin in utero, as a suboptimal intrauterine environment can have a lasting impact on metabolic control [1–5]. A major mechanism by which the effects of an adverse in utero environment appear to be transmitted is by perturbation of the offspring’s DNA methylome [6–8]. Because the DNA methylome is susceptible to both genetic [9–11] and environmental [12, 13] influences, both factors may act during development to program pathways to obesity [14]. Advances in microarray technology have made it feasible for DNA methylation to be quantified at multiple CpG sites across large samples, and has paved the way for epigenome-wide association studies (EWAS) [15].

Birth weight is often used as a surrogate outcome to evaluate the overall quality of the in utero environment. Firstly, both low and high birth weights have been implicated in childhood and adult onset of chronic diseases such as obesity, impaired glucose tolerance, type 2 diabetes mellitus (T2DM) and coronary artery disease [16]. Secondly, modifiable prenatal environmental factors (themselves being determinants of the intrauterine environment), such as maternal obesity and dietary intake, have been linked with birth weight [17]. Thirdly, there is evidence to suggest that birth weight and metabolic diseases share some common genetic determinants [18].

To date, only four epigenome-wide studies have been reported that examined the association between methylation marks in neonate tissues and birth weight [19–22], and all these studies have been conducted on Caucasian populations. All four studies focused largely on the associations between offspring size/adiposity and variations in the neonate DNA methylome. The only study [22] which included genetic information in the analysis had a small sample size. Also, Engel et al. [19], Haworth et al. [22] and Simpkin et al. [20] did not consider the influence of prenatal environments on the identified associations, while Sharp et al. [21] focused exclusively on the contribution of maternal adiposity (pre-pregnancy body mass index (ppBMI) and pregnancy weight gain) to the variation in offspring’s methylome.

We previously reported that *RXRA* promoter methylation in umbilical cord DNA correlates with childhood obesity in replicate cohorts, and that the level of methylation is associated with maternal nutrition in the first trimester [23]. Using a candidate gene approach, we also reported that umbilical cord DNA methylation in the hypoxia inducible factor 3A (*HIF3A*) gene (a gene previously associated with adult adiposity [24]) associates with infant weight and adiposity [25]. It follows that epigenetic alterations associated with the development of adiposity may arise during in utero development. These findings, along with previous findings on the influence of SNPs and in utero environment on the epigenome [14], prompted an EWAS to be performed to comprehensively search for DNA methylome changes in utero in response to variations in prenatal environments and genotype.

In the current study we take a comprehensive approach to understand the genesis of adiposity in early life by interrogating the effects of prenatal environment, genotype and DNA methylation, and we report four important findings. First, we identify prenatal environments that influence birth weight. Second, we report associations between child weight/adiposity (at birth and during early childhood) and polygenic risk score derived from adult adiposity genetic association studies. Third, we find variations in the neonate DNA methylome that associate with birth weight and size/adiposity measures in early childhood. Last, we determine SNPs and specific prenatal environments contributing to this variability in the neonate epigenome. This study is the first large sample size EWAS (N = 987) that assesses the impact of prenatal environment and genetic and epigenetic factors on birth weight and size/adiposity in early childhood. It is also the first neonate EWAS conducted in an Asian population.

## Methods

### Study population

This work is part of the Growing Up in Singapore Towards healthy Outcomes (GUSTO) study, a prospective mother-offspring birth cohort designed to investigate developmental origins of health and disease (DOHaD). The GUSTO cohort has been described previously [26]. Pregnant women of at least 18 years of age and in their first trimester of pregnancy were recruited from the two major public hospitals in Singapore with obstetric services (KK Women’s and Children’s Hospital and the National University Hospital) between 2009 and 2010. Eligible participants were Singaporean citizens, permanent residents, or those who planned to reside in Singapore for the next 5 years, and intended to deliver the baby at the National University Hospital or KK Women’s and Children’s Hospital. They could be of Chinese, Malay or Indian ethnicity, but with homogeneous parental ethnic background. Women who were on chemotherapy or psychotropic drugs were excluded from the study. Interviewer-administered questionnaires were used to assess maternal pre-pregnancy weight, demographics (including maternal age and education) and maternal obstetric and medical history at enrolment. All pregnant women underwent four ultrasound scans during pregnancy to measure fetal growth. Extensive maternal assessments were conducted at 26–28 weeks gestation. All offspring were assessed at birth and at different later time points (3, 6, 9, 12, 15, 18, 24, 36 and 48 months). This study is still active with plans to collect data up to adolescence. This is an interim analysis on an ongoing cohort study. Of the 1177 singleton deliveries, 987 subjects were selected as fulfilling the following inclusion criteria: full-term births with Apgar score ≥ 9, and availability of at least one child weight measurement, infant genotype and methylation data (Additional file 1: Supplementary Figures A1–A3).

### Child characteristics and anthropometry

Child weight and recumbent length/standing height were measured at birth and at nine subsequent time points (3, 6, 9, 12, 15, 18, 24, 36 and 48 months). Child weight was measured using calibrated scales (birth to 18 months: SECA 334 Weighing Scale; 24 to 48 months: SECA 803 Weighing Scale, SECA Corp) and recorded to the nearest gram. Recumbent length (birth, 3, 6, 9, 12, 15, 18, 24 months) was measured using a SECA infant mat (SECA 210 Mobile Measuring Mat, SECA Corp) and recorded to the nearest 0.1 cm. Standing height (36 and 48 months) was measured using a stadiometer (SECA stadiometer 213, SECA Corp) from the top of the child’s head to his or her heels, and recorded to the nearest 0.1 cm. Weight and length/height measurements were taken in duplicates for reliability. BMI was derived as weight (kg) divided by length^2^ (m^2^) at all time points. Subscapular and triceps skinfolds were measured at birth, 18, 24, 36 and 48 months, and taken in triplicate using the Holtain skinfold callipers (Holtain Ltd, Crymych, UK) on the right side of the body, and recorded to the nearest 0.2 mm. Subscapular to triceps skinfold ratio was derived by dividing subscapular skinfold (mm) by triceps skinfold (mm). BMI is used as a proxy for adiposity in the analyses and to be concise, the terms BMI and adiposity have been used interchangeably in this study. Some caution should be exercised in interpreting the findings on BMI, because while BMI is widely accepted as an indirect measure of adiposity, it has its limitations. For example, elevated BMI levels may arise as a result of extra muscle mass or stunted linear growth [27]. We have also included additional analyses using skinfolds to capture adiposity. However, skinfolds were measured at fewer time points and were generally associated with larger measurement error. Gestational age (GA) was determined by ultrasonography in the first trimester. Child sex was taken from the medical records.

### Prenatal environment exposures

An interviewer-administered questionnaire was conducted at 26–28 weeks of gestation to obtain information on occupational activity during pregnancy, alcohol usage before and during pregnancy, and smoking patterns before and during pregnancy. Maternal height and weight were measured during the same time period. Pre-pregnancy weight was self-reported during study recruitment in the first trimester of pregnancy. Gestational weight gain (GWG) was calculated as the difference between the pre-pregnancy and 26–28 week weights. Maternal ppBMI was derived as pre-pregnancy weight divided by height squared. Maternal glucose levels (2-h post-glucose and fasting) were ascertained at 26–28 weeks using an oral glucose tolerance test of 75 g after an overnight fast (8–14 hours). Maternal plasma fatty acids, including n-6 polyunsaturated fatty acids (PUFA), n-3 PUFA, monounsaturated fatty acids (MUFA), and saturated fatty acids, were measured using gas chromatography–mass spectrometry, and expressed as percentage contribution to total plasma phosphatidylcholine (PC) fatty acid. Specifically, plasma lipids were extracted using chloroform–methanol (2:1, v/v) and PC was isolated by solid phase extraction. Fatty acid methyl esters were generated from PC after reaction with methanol containing 2% (v/v) sulfuric acid, extracted into hexane and separated by gas chromatography. Fatty acid methyl esters were identified by comparison with retention times of previous standard runs and quantified using ChemStation software (Agilent Technologies). Maternal micronutrient levels (vitamin D, vitamin B6, vitamin B12, folate, zinc, iron and magnesium) were measured from serum drawn at 26–28 weeks of gestation. Maternal calorie intake at 26–28 weeks gestation was calculated from both 24-h dietary recall and 3-day food diary. Maternal depressive symptoms were assessed using the Edinburgh Postnatal Depression Scale, which was designed and normed expressly for depressive symptoms over the peripartum period [28] and is validated for prenatal screening for depression in Singaporean women [29, 30]. Symptoms of anxiety were assessed using the State–Trait Anxiety Inventory [31], which is a comprehensive and common research tool that measures both stable (trait) and more transient (state) symptoms. Importantly, translation and back-translation of all questionnaires into individual languages, including Chinese, Tamil and Malay, have been performed and validated to ensure consistency to the English version. This study included administration of questionnaires in all three languages according to the language preference indicated by the mother [32]. Birth order and mode of delivery were extracted from hospital medical records. We note that all prenatal exposures/factors listed here contribute to the prenatal environment; to be concise, we have used the term “prenatal environment” to refer to these exposures/factors in the subsequent sections.

### Infant methylation data

Methylation profiling of umbilical cord samples was performed using the Infinium HumanMethylation450 array, following standard protocol, and processed using in-house quality control procedure [33]. Raw methylation beta values were exported from GenomeStudio™. Probes with less than three beads for methylated or unmethylated channel or with detection *P* > 0.01 were set to missing. Probes from sex chromosomes were removed. Colour adjustment and normalisation of Type 1 and 2 probes was performed. Methylation beta values were first converted to M-values before applying COMBAT to remove batch (plate) effects [34], and the batch-corrected methylation values transformed back to beta values. Finally cross-hybridising probes [35, 36], as well as probes where the methylation range (maximum-minimum, excluding outliers) was less than 10%, were excluded, giving a total of 174,211 CpGs for analysis. We did not filter the probes that were annotated to be located within SNPs before analysis. Instead, a post-hoc analysis was performed on the top birth weight-associated CpGs to ensure that (1) no common SNP was located at the CpG and the single base extension, and (2) scatterplots of the methylation values showed a “cloud-like” distribution and not a multi-modal distribution [37, 38].

### Infant genotype data

Genotyping was performed using the Illumina Omniexpress + exome array. Non-autosomal SNPs, SNPs with call rates < 95%, or minor allele frequency < 5%, or those that failed Hardy–Weinberg Equilibrium were excluded from the analysis. Principal components analysis was used to confirm self-reported ethnicity/ancestry. Samples with call rate < 99%, cryptic relatedness and sex/ethnic discrepancies were excluded. Alleles on the positive strand were reported as per the hg19 build of the human genome assembly. After quality control filtering 577,204 SNPs were available for downstream analysis.

### Statistical analysis

The overall analysis framework is summarised in Additional file 1: Supplementary Figure A4 and each analysis is elaborated below. Information on covariates/confounders was available for all 987 infants; where relevant, these variables were adjusted for in the statistical models. These variables included infant ethnicity, infant sex, gestational age and cellular proportions (estimated from DNA methylation data). A complete-case analysis was conducted, i.e. for each model, all infants with complete information for the outcome(s) and predictor(s) were included in the analysis.

#### Prenatal environment influences on birth weight

Linear regression models were used to examine the association of 30 prenatal environment variables with infant birth weight. Eleven of these 30 prenatal environment variables that associated with birth weight were used for subsequent analysis. We first separately studied the association of each prenatal environment variable with birth weight, adjusted for infant sex, ethnicity and GA. This was followed by the association of prenatal environment variables with birth weight, adjusted for each other along with infant sex, ethnicity and GA. We examined the distribution of infant birth weight, and subsequently decided to use a log-transformation on infant birth weight to improve normality and reduce the impact of outliers. Following Gelman [39], binary environment variables were not scaled so that their estimates could be directly interpreted. Since the unscaled binary environment variables generally have a standard deviation (SD) of approximately 0.5, continuous prenatal environment variables were standardised to have a SD of 0.5 (centred and divided by two times SD), so that effect estimates from both continuous and binary prenatal environment variables were comparable. Note that this is different from the Z-score, which is obtained by centring and dividing by one SD. Due to the standardisation of prenatal environment variables and log-transformation on birth weight, effect estimates are interpreted as percentage change in birth weight for a 2 SD increase in prenatal environment variable (for continuous prenatal environment variables), or percentage change in birth weight for comparing two categories of prenatal environment variable (for binary prenatal environment variables).

#### Genetic influences on birth weight

To determine whether genetic variation at loci previously associated with adult adiposity was associated with newborn size/adiposity, polygenic risk score (PRS), or cumulative genetic risk profile, was computed for each infant in the GUSTO cohort using regression coefficients and *P* values for adult BMI reported by the Genetic Investigation of ANthropometric Traits (GIANT) consortium [40]. PRS was computed using the *P* value-informed clumping procedure implemented in PLINK. To reduce the inclusion of SNPs in linkage disequilibrium (LD), two rounds of clumping were performed. We first used a cut-off of R^2^ = 0.5 within a 250-kb window to identify potential index SNPs; in each 250-kb window, the SNP with the smallest *P* value from GIANT was kept while SNPs in LD (R^2^ > 0.5) were removed. Second, to further exclude SNPs in long-range LD with potential index SNPs, the clumping procedure was repeated with a cut-off of R^2^ = 0.2 within a 5-Mbp window. For each individual, the cumulative score was computed by summing the number of score alleles, weighted by the regression coefficients reported by the GIANT consortium. We computed PRS for each ethnic group separately, and at different *P* value thresholds p_T_ for the index SNPs (p_T_ from 10^–10^ to 1). For each ethnic group, PRS was standardised to mean 0 and variance 1 (Z-score) separately. We then regressed PRS against log-transformed child anthropometric measures, adjusted for child sex and GA, for each ethnic group. We examined ethnicity-stratified associations of PRS with child anthropometric outcomes for different *P* value thresholds. For each ethnic group, we selected the *P* value threshold that gave the best-fit score (defined as PRS showing consistent associations with child weight and BMI at multiple time points). This best-fit PRS was then used for subsequent analysis. We did not use other child outcomes (subscapular skinfolds, triceps skinfolds and subscapular:triceps ratio) for evaluating the best-fit score as these outcomes were measured at fewer time points and generally had larger measurement error. We also did not consider child length for evaluating the best-fit score as it did not capture adiposity. However, we report associations between PRS for all child outcomes. For concision, the result sections report only the conclusive findings (child weight and BMI), while the rest (e.g. skinfolds) are provided under Additional file 1: Supplementary File B.

#### Birth weight and neonatal DNA methylome

To interrogate the association between perinatal methylome and birth weight, we performed linear regression of log-transformed birth weight against methylation at each CpG site, adjusted for child sex, GA, ethnicity, cellular proportions and interactions between ethnicity and cellular proportions. Cellular proportions for fibroblasts, B-cells and T-cells were estimated [41] using a cell-specific methylation profile reference panel (accession number EGAD00010000460) [42]. A principal components analysis was performed on the three estimated cellular proportions and the first two principal components adjusted as covariates in all subsequent regression models. Since the associations of estimated cellular proportions with birth weight were ethnicity dependent (data not shown), interaction terms between principal components of cellular proportions and ethnicity were included as covariates in all regression models. For sensitivity analysis, we applied an additional method (surrogate variable analysis) to correct for cellular heterogeneity that did not require a reference-panel of cell-specific methylation profiles [43–45]*.* We further applied genomic control to the *P* values if the genomic inflation factor computed across 174,211 CpGs was greater than 1. A genomic control correction could help correct for residual confounding due to cellular heterogeneity; however, it could also be too conservative, as a global inflation in epigenome-wide *P* values in response to increased adiposity in adults has been previously reported [46] and could be a true biological phenomenon. To adjust for multiple testing across 174,211 CpGs, we report all CpGs associated with birth weight at a false discovery rate (FDR) < 0.05 [47]. This subset of CpGs identified at FDR 0.05 was further investigated below. For CpGs significantly associated with birth weight at FDR 0.05, we also examined if the associations differed among the three ethnic groups by assessing interactions with ethnicity. This analysis was done by regression of log-transformed birth weight against interaction terms between methylation and ethnicity, adjusted for main effects of methylation, main effects of ethnicity, child sex, GA, cellular proportions, and interactions between ethnicity and cellular proportions. Interactions with infant sex were assessed in a similar manner.

#### Genetic and environmental influences on top CpGs

We then characterised the influences of the prenatal environment and SNPs on the variability in methylation at CpGs showing association with birth weight. First, to investigate the influence of the prenatal environment on methylation levels, we regressed methylation at each CpG site against (standardised) prenatal environment variables, adjusting for child sex, GA, ethnicity, cellular proportions and interactions between ethnicity and cellular proportions. To adjust for multiple testing across eight CpGs and 11 prenatal environments, a CpG was defined to be influenced by the prenatal environment if the most significant association with the prenatal environment variables had genomic control-adjusted *P* < 0.05/(8 × 11) ~ 5 × 10^–4^. Genomic inflation factor was computed for each prenatal environment across all 174,211 variable CpGs, and genomic control was applied if the inflation factor for the variable was above 1. This simple Bonferroni correction for multiple testing across the 11 prenatal environments was likely to be conservative as the 11 prenatal environments were associated with one another (for example, a mother who smokes during pregnancy is highly likely to be smoking before pregnancy). FDR was not used for multiple testing adjustments here and for the top CpGs and offspring size/adiposity in early childhood because of the relatively small number of tests (88 and 360, respectively) and dependency between the tests [48, 49]. For each CpG, we also report the prenatal environment variable that showed the strongest association (smallest *P* value) with the CpG.

Second, to interrogate the influence of SNPs on methylation levels, we regressed each CpG against *cis*-SNPs (defined here as SNPs on the same chromosome as CpG), using an additive genetic model, adjusted for child sex, GA, ethnicity, cellular proportions, and interactions between ethnicity and cellular proportions. For SNPs where the minor homozygote genotype group had ≤ 50 individuals, the minor homozygote and heterozygote genotype groups were combined (dominant genetic model). A total of approximately 5 × 10^5^ CpG-SNP tests were conducted, corresponding to testing eight CpGs across 8,392 to 47,298 *cis*-SNPs for each CpG (each CpG was tested against 8,392 to 47,298 *cis*-SNPs depending on the chromosome of the CpG). A CpG was defined to be influenced by the genotype (SNPs) if the most significant association between the CpG and *cis*-SNPs attained *P* < 1 × 10^–7^, the Bonferroni threshold to maintain a family-wise Type 1 error rate of 0.05 across approximately 5 × 10^5^ tests.

#### Top CpGs and offspring size/adiposity in early childhood

Finally, we examined whether these methylation marks at birth were associated with offspring weight in early childhood (3–48 months) and offspring length and adiposity (BMI, subscapular skinfold, triceps skinfold and subscapular:triceps ratio) from birth to 48 months. We also examined BMI change in early childhood (calculated as the difference between BMI Z-score at 48 months and birth), where BMI Z-score at birth and 48 months were calculated using WHO child growth charts. Child anthropometric measures (except BMI change) were log-transformed to improve normality and reduce the impact of outliers. Each offspring anthropometric measure at each assessment time point was analysed separately. This was done by linear regression of (log-transformed) anthropometric measures at each time point against the methylation at each CpG site, adjusted for child sex, GA, ethnicity, cellular proportions and interactions between ethnicity and cellular proportions. To account for multiple testing across the eight CpGs and 45 child size/adiposity measures, a CpG would be associated with offspring size/adiposity if the genomic control-adjusted *P* < 0.05/(8 × 45) = 1 × 10^–4^. The genomic inflation factor was computed for each offspring anthropometry measure across all 174,211 CpGs, and genomic control was applied if the inflation factor for the anthropometry outcome was above 1. This simple Bonferroni correction for multiple testing across different size/adiposity measures was likely to be extremely conservative as the 45 size/adiposity measures were strongly associated with each other. For concision, the result sections describe only the conclusive findings, while the rest are reported in Additional file 1: Supplementary File F.

#### Multiple testing corrections

We used different multiple testing methods (FDR vs. Bonferroni) at different analysis steps in the sections above. The reason for the use of different methods was due to the vastly different number of tests to be adjusted for multiple testing in each analysis step. To adjust for multiple testing across 174,211 CpGs in birth weight and neonatal DNA methylome, we used FDR. For the environmental influences on top CpGs and for the top CpGs and offspring size/adiposity in early childhood sections, FDR could not be used for multiple testing adjustments because of the relatively small number of tests (88 and 360, respectively) and dependency between the tests [48, 49]. Instead, we used Bonferroni threshold in order to maintain a family-wise Type 1 error rate of 0.05 at each analysis step.

### Accessing DNA methylation data

The infant methylation data analyzed in the current study is available as Additional files 2 and 3.

## Results

### Birth weight is associated with 11 prenatal environments

This analysis used 987 of 1177 singleton deliveries in the GUSTO cohort study. The subject selection criterion included live singleton term births with Apgar score ≥ 9, and availability of anthropometric measures, covariates/confounder information, as well as genotyping and methylation data for all subjects (Additional file 1: Supplementary Figures A1–A3). Summary statistics of these 987 mother-offspring participants are provided in Tables 1 and 2; 58%, 17% and 25% of the participants were from Chinese, Indian and Malay ethnicity, respectively; and 52% of the infants were male. The number of children with age- and sex-specific BMI Z-score exceeding 2 and 3 at each time point are reported in Additional file 1: Supplementary Table A1. The number of mothers who were underweight, normal weight, overweight and obese before pregnancy is reported in Additional file 1: Supplementary Table A2. Using non-Asian BMI cut-offs, 12%, 64%, 17% and 7% of the mothers were underweight, normal weight, overweight and obese, respectively, before pregnancy. When Asian-specific BMI cut-offs were used, more women were classified as being overweight (22%) and obese (14%) before pregnancy.

**Table 1:** Offspring characteristics of the GUSTO cohort studied in the analysis

		Time point	N (%)	Mean (SD)
Ethnicity	Chinese	Delivery	570 (58%)	
	Malay		247 (25%)	
	Indian		170 (17%)	
Child sex	Male		517 (52%)	
	Female		470 (48%)	
Gestational age (weeks)			987	39 (1)
Weight (g)		Delivery	959	3130.5 (380.9)
		3 months	904	6150.6 (778.7)
		6 months	864	7717.1 (914.3)
		9 months	829	8615.0 (1001.4)
		12 months	846	9380.2 (1078.6)
		15 months	851	10086.2 (1164)
		18 months	804	10742.4 (1298.7)
		24 months	818	11981.6 (1552.8)
		36 months	824	14249.8 (2028.2)
		48 months	718	16442.1 (2692.4)
Length/height (cm)		Delivery	959	48.7 (1.8)
		3 months	904	60.9 (2.4)
		6 months	868	67.1 (2.7)
		9 months	830	71.6 (2.8)
		12 months	848	75.4 (3.1)
		15 months	843	78.9 (3.2)
		18 months	689	82.1 (3.3)
		24 months	718	87.6 (3.6)
		36 months	817	94.8 (3.8)
		48 months	716	102.3 (4.2)
Body mass index (kg/m^2^)		Delivery	959	13.2 (1.2)
		3 months	904	16.5 (1.6)
		6 months	864	17.1 (1.6)
		9 months	829	16.8 (1.5)
		12 months	845	16.5 (1.4)
		15 months	843	16.2 (1.4)
		18 months	687	15.9 (1.3)
		24 months	718	15.5 (1.4)
		36 months	817	15.8 (1.5)
		48 months	716	15.6 (1.8)
Subscapular skinfold (mm)		Delivery	959	5.0 (1.2)
		18 months	671	6.4 (1.4)
		24 months	757	6.4 (1.6)
		36 months	792	6.6 (1.9)
		48 months	674	6.8 (2.7)
Triceps skinfold (mm)		Delivery	960	5.5 (1.3)
		18 months	709	8.6 (1.7)
		24 months	733	8.8 (1.8)
		36 months	786	9.3 (2.3)
		48 months	684	9.8 (2.9)
Subscapular:Triceps		Delivery	959	0.9 (0.2)
		18 months	646	0.8 (0.1)
		24 months	722	0.7 (0.1)
		36 months	780	0.7 (0.1)
		48 months	671	0.7 (0.1)

**Table 2:** Maternal characteristics of the GUSTO cohort studied in the analysis

		Time point	N (%)	Mean (SD)
Pre-pregnancy BMI (kg/m^2^)		Self-reported at first clinic visit	906	22.7 (4.4)
Gestational weight gain (kg)		26–28 weeks gestation	902	8.7 (4.7)
Maternal height (m)			971	158.3 (5.6)
Fasting glucose (mmol/L)			920	4.3 (0.5)
2-h post-glucose (mmol/L)			920	6.5 (1.5)
n-6 PUFA (%)			863	34.2 (3.3)
n-3 PUFA (%)			863	6.4 (1.8)
MUFA (%)			863	13.6 (2.3)
SFA (%)			863	45.8 (3.3)
EPDS score			955	7.4 (4.4)
STAI state score			957	33.8 (10.0)
STAI trait score			957	35.7 (9.6)
Caloric intake 3-day food diary (kcal)			550	1871.2 (476.3)
Caloric intake 24-h recall (kcal)			960	1843.6 (550.6)
Parity	>0	Delivery	536 (54%)	
	0		451 (46%)	
Maternal age (years)	≥35	Self-reported at first clinic visit	251 (25%)	
	<35		736 (75%)	
Smoking before pregnancy	Yes	Interviewer-administered questionnaire at 26–28 weeks gestation	121 (12%)	
	No		855 (88%)	
Smoking during pregnancy	Yes		24 (2%)	
	No		951 (98%)	
Plasma vitamin D	>50 nmol/L	26–28 weeks gestation	718 (87%)	
	≤50 nmol/L		108 (13%)	
Plasma folate	≥6 ng/mL		774 (90%)	
	<6 ng/mL		90 (10%)	
Plasma vitamin B12	≥300 pg/mL		373 (43%)	
	<300 pg/mL		492 (57%)	
Plasma vitamin B6	<20 nmol/L		137 (16%)	
	≥20 nmol/L		727 (84%)	
Plasma iron	≥560 μg/L		403 (92%)	
	<560 μg/L		36 (8%)	
Plasma zinc	≥700 μg/L		417 (95%)	
	<700 μg/L		22 (5%)	
Plasma magnesium	≥18.25 mg/L		304 (69%)	
	<18.25 mg/L		135 (31%)	
IVF birth	Yes	Self-reported at first clinic visit	69 (7%)	
	No		918 (93%)	
Maternal education (years)	≥12		596 (61%)	
	<12		379 (39%)	
Working during pregnancy	Yes	Interviewer-administered questionnaire at 26–28 weeks gestation	681 (70%)	
	No		297 (30%)	
Alcohol use before pregnancy	Yes		338 (35%)	
	No		636 (65%)	
Alcohol use during pregnancy	Yes		19 (2%)	
	No		938 (98%)	

*BMI* body mass index, *EPDS* Edinburgh Postnatal Depression Scale, *IVF* in vitro fertilisation, *MUFA* monounsaturated fatty acids, *PUFA* polyunsaturated fatty acids, *SFA* saturated fatty acids, *STAI* State–Trait Anxiety Inventory

**Fig. 1:**
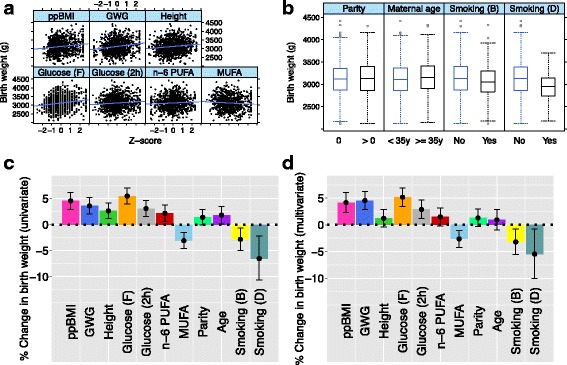
Prenatal environment influences on birth weight. **a** Scatterplots of birth weight (vertical axis) against significantly associated continuous prenatal environment variables (horizontal axis). **b** Boxplots of birth weight (vertical axis) against significantly associated binary prenatal environment variables (horizontal axis). **c** Univariate association between birth weight and each significantly associated prenatal environment variable, adjusted for infant sex, ethnicity and gestational age. Point estimates (height of bars) and 95% confidence intervals (top and bottom whiskers), show percentage change in birth weight for two standard deviations increase in continuous prenatal environment variable, or for comparing the two categories of binary prenatal environment variables. **d** Multivariate association between birth weight and significantly associated prenatal environment variables, adjusted for infant sex, ethnicity, gestational age and for each other. Point estimates (height of bars) and 95% confidence intervals (top and bottom whiskers), show percentage change in birth weight, for two standard deviations increase in a continuous prenatal environment variable, or for comparing the two categories of binary prenatal environment variables

We assessed 30 prenatal environment variables for association with birth weight (Additional file 1: Supplementary Table A3). Of the 30 prenatal environment variables analysed (Tables 1 and 2), infant birth weight was associated with maternal ppBMI, maternal GWG, maternal height, maternal glucose levels (fasting and 2-h post-75 g-glucose challenge), maternal plasma n-6 PUFA and MUFA levels at 26 weeks gestation, maternal age, and maternal smoking before and during pregnancy (Fig. 1; Additional file 1: Supplementary Tables A3 and A4; *P* < 0.05). There was also a suggestive association with parity (Fig. 1; Additional file 1: Supplementary Tables A3 and A4; *P* = 0.059). Greater maternal adiposity (ppBMI and GWG), height, glucose levels (fasting and 2-h post-glucose), n-6 PUFA levels, age and parity were associated with higher birth weight, while higher MUFA levels and maternal smoking (before and during pregnancy) were associated with lower birth weight. Birth weight changed by 2.2–5.5% for every 2 SD change in maternal adiposity (ppBMI and GWG), height, glucose levels (fasting and 2-h post-glucose) or FA levels (n-6 PUFA and MUFA). The effect sizes for parity (non-first born vs. first born), maternal age (≥35 years vs. < 35 years) and smoking (yes vs. no) were similar and ranged from 1.4% to 6.5% (Fig. 1c; Additional file 1: Supplementary Table A3). Seven of 11 of these prenatal environment variables, including maternal adiposity (ppBMI and GWG), glucose levels (fasting and 2-h post-glucose), FA levels (n-6 PUFA and MUFA), and smoking during pregnancy, also showed association with child BMI at birth (*P* < 0.05; Additional file 1: Supplementary Figure A6). Consistent with earlier findings [50, 51], maternal ppBMI, GWG and glucose levels were also significantly associated with both child weight and BMI at 48 months of age (Additional file 1: Supplementary Figures A5–A8). For subsequent analyses on the associations of prenatal environment with neonate DNA methylation, we restricted the analyses to the 11 birth weight associated prenatal environment variables shown in Fig. 1. We note that these 11 prenatal environments are not distinct/independent of each other, for example, a mother who smokes during pregnancy is highly likely to have been smoking before pregnancy.

### Birth weight and early childhood adiposity were significantly associated with polygenic risk score derived from adult population studies

**Fig. 2:**
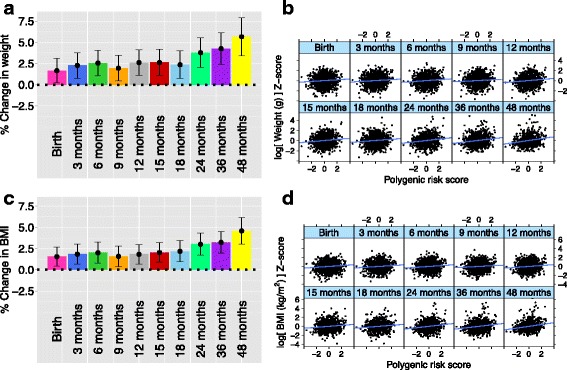
Genetic influences on birth weight: Associations of child weight (**a** and **b**) and body mass index (**c** and **d**) at different time points with best-fit polygenic risk score (PRS). Best-fit PRS for Chinese, Malay and Indian ethnic groups used clumping *P* value thresholds p_T_ = 0.5, 0.1 and 10^–4^, respectively. PRS was standardised to mean zero and unit variance within each ethnic group. Left panel (**a** and **c**) shows point estimates (height of bars) and 95% confidence intervals (top and bottom whiskers), for percentage change in child outcome, for a 2 SD increase in PRS, adjusted for child sex, gestational age and ethnicity. Analysis was done by linear regression of log-transformed child anthropometric outcome at each time point against PRS, adjusted for child sex, gestational age and ethnicity. Right panel (**b** and **d**) shows scatterplot of standardised (mean zero and unit variance) log-transformed child outcome (vertical axis) against PRS (horizontal axis)

To interrogate if the genetic variation at loci previously associated with adult adiposity was associated with newborn size/adiposity, PRS or cumulative genetic risk profile was constructed using genetic variants previously reported to be associated with adult BMI by the GIANT consortium [40]. This PRS showed a significant association with birth weight, supporting an overlap in the genetic factors contributing to birth weight and adult adiposity (Fig. 2; Additional file 1: Supplementary Figure B1 and Supplementary Table B1). Birth weight increased by 1.6% for every 2 SD increase in PRS (Fig. 2a; Additional file 1: Supplementary Table B1). The association of PRS with birth weight remained even after adjusting for the 11 prenatal environment variables (Additional file 1: Supplementary Table A5 and Supplementary Figure A9), and the association of the prenatal environment variables with birth weight was not PRS dependent (Additional file 1: Supplementary Table A6), indicating the independent influences of genotype and prenatal environment on birth weight; 18% of the total variation in birth weight was explained by infant sex, ethnicity and GA, while an additional 14% was explained by 11 prenatal environment variables and PRS together. An association between PRS and child BMI at birth was also observed (Fig. 2b) and PRS was longitudinally associated with weight and BMI in early childhood (Additional file 1: Supplementary Figure B1 and Supplementary Table B1). This longitudinal association of PRS with weight and BMI from 3 to 48 months of age remained after adjustment for birth weight or BMI (Additional file 1: Supplementary Figure B2).

### Birth weight was significantly associated with methylation at eight CpGs within seven gene loci

DNA from umbilical cord tissue of the 987 neonates was interrogated on Infinium HumanMethylation450 BeadChip arrays. A total of 174,211 CpGs were identified to vary in methylation by more than 10% across the subjects. These CpGs were more likely to be located in open seas and intronic/intergenic regions (Additional file 1: Supplementary Figure C1). An EWAS on birth weight was performed using these variably methylated CpGs, and adjusted for child sex, GA, ethnicity, cellular proportions and interactions between ethnicity and cellular proportions. Methylation levels at eight CpGs were identified to be significantly associated with birth weight at a FDR of 0.05 (Table 3; Additional file 1: Supplementary Figures C2 and C3). Among them, six CpGs were located within the protein coding genes: (1) 5’-UTR of Ankyrin-3 (*ANK3*; *P* = 4.6 × 10^–8^); (2) 3’-UTR of Cyclin-Dependent Kinase Inhibitor 2B (*CDKN2B*; *P =* 4.9 × 10^–8^); (3) intron of Immunoglobulin Superfamily, DCC Subclass, Member 4 (*IGDCC4*; *P =* 1.6 × 10^–7^); (4) intron of Prolyl 4-hydroxylase, Alpha Polypeptide III (*P4HA3*; *P* = 4.0 × 10^–7^); (5) intron of Calcium Channel, Voltage-Dependent, T Type, Alpha 1G Subunit (*CACNA1G*; *P* = 1.2 × 10^–6^); and (6) intron of Zinc Finger Protein 423 (*ZNF423*; *P* = 1.9 × 10^–6^), while the remaining two CpGs mapped to the non-coding gene *MIRLET7BHG* (*P* = 9.9 × 10^–7^ and *P* = 2.2 × 10^–6^).

**Table 3:** Methylome-CpGs associated with birth weight at a false discovery rate of 0.05

CpG	CHR	POS	IQR	Est	95% CI	*P*	Gene	Annotation
cg00510507	10	61900413	8.4	4.9	(3.5 to 6.2)	4.6 × 10^–8^	*ANK3*	5’ UTR
cg08390209	9	22005563	6.6	7.1	(5.1 to 9.0)	4.9 × 10^–8^	*CDKN2B*	3’ UTR
cg23671997	15	65677753	4.6	9.2	(6.5 to 12)	1.6 × 10^–7^	*IGDCC4*	Intron
cg14300531	11	73969506	9.6	–3.9	(–5.0 to –2.8)	4.0 × 10^–7^	*P4HA3*	Intron
cg25685359	22	46473721	8.8	–3.7	(–4.8 to –2.6)	9.9 × 10^–7^	*MIRLET7BHG*	Non-coding
cg22383874	17	48670670	4.8	7.6	(5.2 to 10)	1.2 × 10^–6^	*CACNA1G*	Intron
cg02729344	16	49888237	6.6	6.8	(4.7 to 9.0)	1.9 × 10^–6^	*ZNF423*	Intron
cg25487405	22	46473039	5.5	–5.6	(–7.2 to –3.9)	2.2 × 10^–6^	*MIRLET7BHG*	Non-coding

Eight CpGs were significantly associated with birth weight at a false discovery rate (FDR) of 0.05. The eight CpGs mapped to seven loci (two CpGs mapped to *MIRLET7B*HG). Regression coefficients (Est), 95% confidence intervals (CI) and *P* values are reported as percentage change in birth weight for 10% increase in percent methylation. Interquartile range (IQR), chromosome (CHR) and position (POS) of CpG are also shown. Analysis was done by linear regression of log-transformed birth weight against methylation at each CpG site, adjusted for child sex, gestational age, ethnicity, cellular proportions and interactions between ethnicity and cellular proportions

Variability in methylation at these seven gene loci (eight CpGs) was modest with an interquartile range (IQR) of 4.6% to 9.6%. DNA methylation levels at five of seven loci (five CpGs) were positively associated with birth weight, while DNA methylation at the remaining two loci (three CpGs) were negatively associated with birth weight. The effect sizes were modest, with a 3.7–9.2% change in birth weight associated with a 10% increase in methylation (corresponding to approximately 0.4–2 IQR). Together, these eight CpGs accounted for an additional 9.5% of the total variation in birth weight, in addition to the 32% accounted by infant sex, ethnicity, GA, 11 prenatal environments and PRS. Sensitivity analysis using a reference-free method to adjust for cellular heterogeneity gave similar results (Additional file 1: Supplementary Table C1). The associations between birth weight and methylation at these sites did not depend on ethnicity (Additional file 1: Supplementary Table C2) or infant sex (Additional file 1: Supplementary Table C3). For subsequent analyses, we used all seven loci (eight CpGs) identified at FDR < 0.05.

### Methylation levels at three of the seven birth weight-linked loci were significantly associated with prenatal environment

**Fig. 3:**
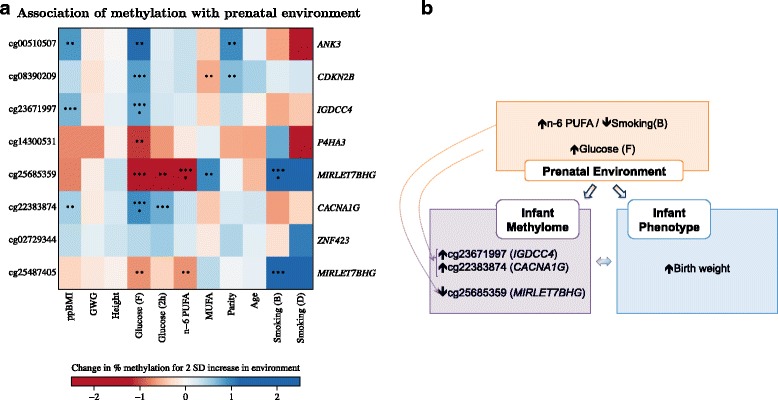
Influence of prenatal environment on methylome at birth. **a** Associations of DNA methylation at birth with prenatal environment. Colour in heatmap represents regression coefficients for associations between methylation and each prenatal environment variable. Each row represents a CpG and each column represents a prenatal environment variable. With increasing magnitudes, colour changes from white to red (for negative coefficients) or from white to blue (for positive coefficients). Asterisks within each square represent *P* values for associations between methylation and each prenatal environment variable (*P* < 5 × 10^–8^ is represented with eight asterisks, 5 × 10^–8^ ≤ *P* < 5 × 10^–7^ is represented with seven asterisks, 5 × 10^–3^ ≤ *P* < 5 × 10^–2^ is represented with two asterisks, *P* ≥ 5 × 10^–2^ is represented with a blank square). Analysis was done by linear regression of methylation at each CpG site against each prenatal environment variable, adjusted for child sex, gestational age, ethnicity, cellular proportions and interactions between ethnicity and cellular proportions. Regression coefficients and *P* values are reported as an increase in percent methylation for a 2 SD increase in continuous prenatal environment variable, or for comparing the two categories of binary prenatal environment variables. **b** Flow chart summarises associations between birth weight, methylation and prenatal environment for three CpGs (three loci) influenced by the prenatal environment. A CpG was defined to be influenced by the prenatal environment if the most significant association between the CpG and prenatal environment attained a *P* value of < 5 × 10^–4^ the Bonferroni threshold to maintain a family-wise Type 1 error rate of 0.05 across approximately 100 tests (8 CpGs x 11 prenatal environment variables). Directions in arrows indicate temporal sequence, measurements obtained at the same time are indicated with two-headed arrows

We interrogated the contribution of individual prenatal environments on variability in the epigenome at these seven loci (eight CpGs). Methylation levels at three of seven loci (*IGDCC4*, *MIRLET7BHG*, *CACNA1G*) were significantly associated with the prenatal environment after adjusting for multiple testing (Fig. 3; Additional file 1: Supplementary Table D1; *P* < 5 × 10^–4^). Methylation levels at cg25685359 (*MIRLET7BHG*) showed a significant inverse association with maternal n-6 PUFA levels (Fig. 3; *P* = 4.2 × 10^–4^), and a significant positive association with maternal smoking before pregnancy (Fig. 3; *P* = 2.3 × 10^–4^). Methylation levels at cg25487405, which also mapped to *MIRLET7BHG*, showed modest associations (*P* < 0.05) with these two prenatal environment variables, though the associations did not survive multiple testing adjustments. The directionality of the associations between methylation and prenatal environments is consistent (Fig. 3) as cg25685359 (*MIRLET7BHG*) showed a negative association with birth weight (Table 3), and birth weight was positively associated with maternal n-6 PUFA levels but negatively associated with maternal smoking (Fig. 1).

Methylation at cg23671997 (*IGDCC4*) showed a significant positive association with maternal fasting glucose levels (Fig. 3; *P* = 2.7 × 10^–4^), and it also showed positive association with maternal ppBMI (*P* = 8.1 × 10^–4^). Likewise, methylation at cg22383874 (*CACNA1G*) was significantly and positively associated with maternal fasting glucose levels (Fig. 3; *P* = 1.7 × 10^–4^), and was also positively associated with maternal ppBMI (*P* = 2.9 × 10^–2^) and maternal 2-h post-glucose levels (*P* = 4.2 × 10^–3^). The directionality of associations between cg23671997 (*IGDCC4*) and cg22383874 (*CACNA1G*) and the prenatal environments is consistent (Fig. 3), as methylation levels at both CpGs were positively associated with birth weight (Table 3), and birth weight was positively associated (Fig. 1) with maternal adiposity-related influences (ppBMI, fasting and 2-h post-glucose levels at mid-pregnancy). After adjustment for maternal ppBMI, the associations of cg23671997 (*IGDCC4*) and cg22383874 (*CACNA1G*) with maternal fasting maternal glucose levels were similar but slightly reduced (*P* = 2.7 × 10^–4^ vs. *P* = 2.3 × 10^–3^ for cg23671997; *P* = 1.7 × 10^–4^ vs. *P* = 4.0 × 10^–4^ for cg22383874; Fig. 3a vs. Additional file 1: Supplementary Figure D1).

### Methylation levels at three of the seven birth weight linked loci were significantly associated with SNPs

To investigate the influence of genetic polymorphisms on methylation at the seven birth weight associated loci (eight CpGs), we regressed each CpG against all *cis*-SNPs (SNPs on the same chromosome as the CpG). Three loci were significantly associated with *cis*-SNPs after adjusting for multiple testing (Additional file 1: Supplementary Table E1). These three loci included *P4HA3*, *ZNF423* and *MIRLET7BHG* (only one of the two *MIRLET7BHG* CpGs was significantly associated with SNPs). The CpG-SNP distances ranged from 12 to 168 kb (Additional file 1: Supplementary Table E1). For these three CpG-SNP pairs, the association of methylation with birth weight (effect sizes and *P* values) was similar with and without adjustment for genotype at the SNP, and the genotype at the SNP was not associated with birth weight (Additional file 1: Supplementary Table E2). Finally, we also investigated if the PRS was associated with methylation levels at these eight CpGs, and also if the PRS moderates the associations between methylation and birth weight/environment*,* but no significant associations were observed (Additional file 1: Supplementary Tables E3–E5).

### Methylation levels at six of the seven birth weight linked loci predicted offspring size/adiposity at 48 months

**Fig. 4:**
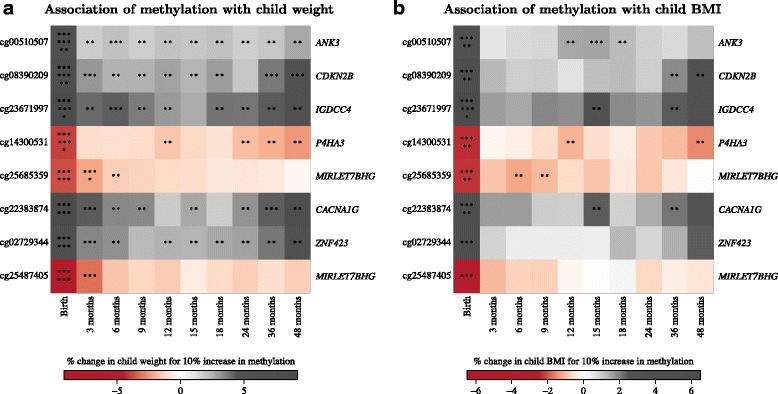
Influence of methylome at birth on adiposity outcomes in early childhood: Associations of child weight (**a**) and body mass index (**b**) at different time points with DNA methylation at birth. Colour in heatmap represents regression coefficients for associations between child anthropometric outcome and methylation. Each row represents a CpG and each column represents a time point. With increasing magnitudes, colour changes from white to red (for negative coefficients) or from white to grey (for positive coefficients). Asterisks within each square represent *P* values for associations between child anthropometric outcome and methylation (*P* < 5 × 10^–8^ is represented with eight asterisks, 5 × 10^–8^ ≤ *P* < 5 × 10^–7^ is represented with seven asterisks, 5 × 10^–3^ ≤ *P* < 5 × 10^–2^ is represented with two asterisks, *P* ≥ 5 × 10^–2^ is represented with a blank square). Analysis was done by linear regression of log-transformed child anthropometric outcome at each time point against methylation at each CpG site, adjusted for child sex, gestational age, ethnicity, cellular proportions and interactions between ethnicity and cellular proportions. Regression coefficients and *P* values are reported as percentage change in child anthropometric outcome for 10% increase in percent methylation

Methylation levels at all seven loci (eight CpGs) showed association with child weight in at least one time point in early childhood (3–48 months), even though these associations did not survive multiple testing adjustments (Fig. 4a; *P* < 0.05). The effect sizes (associations between methylation and child weight) were either (1) strongest at birth and decreased from 3 to 48 months, or (2) strong at birth, decreased initially, and then increased from 18 to 48 months, or (3) strongest at birth, but remained the same (approximately) from 3 to 48 months (Fig. 4a). Methylation levels at six of seven loci (six CpGs) were also significantly associated (*P* < 1 × 10^–4^) with BMI at birth (the remaining two CpGs showed suggestive associations; *P* < 0.005); the change in effect sizes of BMI with child age showed a similar pattern as that of child weight (Fig. 4b). At age 48 months, methylation levels at six of seven loci (six CpGs) and two of seven loci (two CpGs) showed moderate associations with child weight and BMI, respectively (*P* < 0.05). The associations between neonate methylation and child size/adiposity in early childhood (3–48 months) were not independent of birth weight (data not shown). Methylation levels at cg25685359 (*MIRLET7BHG*) showed a suggestive association with BMI change in early childhood (Additional file 1: Supplementary Table F1), where BMI change was calculated as the difference between age- and sex-specific Z-score at 48 months and birth; this association did not survive adjustment for birth weight either (*P* > 0.05).

## Discussion

We have demonstrated that genetic, epigenetic and prenatal environmental factors are linked to offspring size and adiposity at birth and in early childhood. Firstly, we identified individual prenatal environmental influences on birth weight; we have previously reported that some of these prenatal environment variables (maternal ppBMI, GWG and glucose levels) continued to associate with offspring size and adiposity in early childhood [50, 51]. Secondly, genetic variation, as captured by PRS, not only influenced birth weight, but also child size and adiposity up to 48 months of age, independent of birth weight. The PRS was constructed using adiposity-linked genetic risk variants previously reported in an adult population. The association of adult adiposity risk score with size and adiposity in our paediatric population indicates that the effects of genetic risk variants can be detected as early as birth. This finding is also in confirmation with the earlier study that reported an association between newborn weight and adiposity with adult adiposity-derived PRS [52]. Thirdly, neonatal methylation levels at seven loci were associated with birth weight. At six of the seven loci, there was suggestive evidence that the associations continued to persist up to 48 months of age. Among them, two of the loci (*CDKN2B*/*P4HA3*) also showed suggestive association with child BMI at 48 months. Even though the associations in early childhood did not survive multiple testing corrections, these CpGs still hold potential as biomarkers of adverse metabolic trajectory as the prevalence of obesity increases with age and might become more apparent later in the life-course. Lastly, methylation levels at three of seven loci associated with birth weight (*IGDCC4*, *MIRLET7BHG*, *CACNA1G*) also showed significant associations with the prenatal environment; however, similar analyses with childhood weight and adiposity measures showed suggestive associations. Together, these findings provide evidence that birth weight is influenced by both genetic and prenatal environment factors, possibly acting through different mechanisms, either by altering the epigenome (evidenced by CpGs that were associated with prenatal environment and/or SNPs) or independently of the epigenome (e.g. the PRS).

Notably, four of seven methylation loci were located in coding genes (*ANK3*, *CDKN2B*, *CACNA1G*) and the miRNA let-7b host gene (*MIRLET7BHG*) that have been previously implicated in metabolic disorders in human adults and animal model systems. *ANK3* encodes a protein from ankyrin family, and ankyrins have been associated with age dependent adiposity and insulin resistance in a rat model system [53]. *CDKN2B* is known to be involved in metabolic processes since it is highly expressed in subcutaneous adipose tissue, and its expression alters with energy balance (higher expression in obese subjects and down-regulated expression during calorie restriction-induced weight-loss) [54]. Furthermore, genetic variants near the *CDKN2A/B* 9p21.3 locus were previously found to be associated with risk for CVD and T2DM in adults [55]. T-type calcium channels are implicated in maintaining body weight in a rat model, where the administration of *CACNA1G* antagonists to obese rodents results in reduced body weight and fat mass, and increased lean muscle mass [56]. MicroRNA let-7B, transcribed from the *MIRLET7BHG* host gene, belongs to the let-7 family of miRNA that is known to play an important role in adipocyte differentiation (3T3-L1 mouse cells) by targeting *HMGA2*, a transcription factor that regulates growth and proliferation [57, 58]. Furthermore, transgenic mouse experiments have shown that let-7 is a potent regulator of glucose metabolism and peripheral insulin receptors, by targeting insulin-like growth factor 1 (*Igf1r*), insulin receptor (*Insr*) and insulin receptor substrate-2 (*Irs-2*) in skeletal muscle and liver tissues [59]. Let-7 is also a potential biomarker for metabolic disease. In a human interventional study reducing the glycemic load in the diet of healthy premenstrual women, let-7b was the most dramatically altered miRNA, with nearly an eightfold increase of plasma let-7b after 12 months [60].

As mentioned earlier, some of these loci also showed association with either the prenatal environment (*MIRLET7BHG*, *IGDCC4*, *CACNA1G*) or suggestive association with child BMI at age 48 months (*CDKN2B*, *P4HA3*). Collectively, our findings fit within the paradigm of epigenetic mediation in the DOHaD hypothesis. According to the DOHaD hypothesis, the predisposition to adulthood diseases is primed in utero by specific antenatal environments [6], and the mechanistic underpinnings of this phenomenon includes alterations in the epigenome [6]. Here, our discovery of an altered neonatal epigenetic profile at metabolism-linked gene loci and its associations with prenatal environment and the onset of adiposity in utero fit with this paradigm. However, we note that the longitudinal contributions of prenatal environment and the associated changes in the methylome were observed to be moderate for childhood adiposity. Obesity is a complex multifactorial disease that is responsive to environmental changes. Likewise, the epigenome is a modifiable factor and sensitive to developmental and environment cues. In the future, it would be critical to test how these prenatal environment induced changes in the child’s methylome interact or alter with the postnatal environment and developmental changes. It is evident that the associations of methylation (at birth weight-linked loci) with child weight and adiposity either (1) stayed strongest at birth and declined by 48 months, or (2) stayed strong at birth, decreased initially, and then increased from 18 to 48 months, or (3) were strong at birth, but remained the same (approximately) from 3 to 48 months (Fig. 4a and b). These observations very well indicate that epigenetic programing of obesity in early life is dynamic, and can either weaken, strengthen, or stay unchanged with time. Hence, it is possible that some of these epigenetic variations acquired at birth will either become benign, or stay active and become more detrimental later in the life-course. This is further supported by published literature which shows that childhood obesity increases with age; the prevalence of childhood obesity among children aged 7–11 years is almost double than that of children aged 2–6 years [61]. Evaluation of these candidate loci for subject risk stratification or obesity prevention requires further work to examine how DNA methylation levels at these loci changes with age and environmental exposures during childhood.

This study has several strengths, including its prospective and longitudinal study design with a relatively large sample size. The previous three birth weight EWAS [19–21] that had comparable sample sizes did not incorporate genetic or extensive prenatal environment information. Also, the longitudinal offspring anthropometric measures allowed us to study the association of perinatal methylation with both birth and postnatal outcomes for up to 48 months of age. Simpkin et al*.* [20] and Sharp et al*.* [21] also examined adiposity measures (and methylation measures) in childhood and adolescence, but did not provide detailed information in early childhood. Additionally, our study population is comprised of three major Asian ethnic groups that make up more than 40% of the world’s population, while previous investigations were conducted primarily among Caucasian participants. On examination of the CpGs previously reported to be associated with birth weight [19–22], cg04521626, which mapped to the phospholipase D2 (*PLD2*) gene, was statistically significant after adjustment for multiple testing in our cohort (Additional file 1: Supplementary Table C4). For other CpGs that we could not replicate in our study, deviations from previous findings could be due to the underlying differences in the populations examined (different genetic and/or prenatal environment influences in different ethnic groups). Deviations could also be due to differences in tissues assayed (cord tissue vs. cord blood) as DNA methylation is cell type-specific, and cord tissue and cord blood have different cellular composition and cell lineages. For example, cord tissue contains stromal cells from mesenchymal stem cell lineage [62, 63], while cord blood contains mostly cell types from hematopoietic stem cell lineage. This further suggests that neonate EWAS findings may be ethnicity and/or tissue-specific. Cross-tissue/cross-population studies are needed to generalise the findings to other tissues/populations. Additionally, cross-tissue comparisons will enable us to distinguish between common and tissue-specific signals.

There are limitations of this study. First, residual confounding is a concern in any epidemiological investigation. In the context of EWAS, one of the major sources stems from cellular heterogeneity of the tissue being surveyed, as different cell types can have distinct methylation profiles. Cord tissue, like other infant tissues examined in a neonate EWAS, is heterogeneous in its cellular content and consists of stromal, epithelial and endothelial cells (and possibly cord blood contamination) [62, 63]. To combat the issue of cellular heterogeneity, we employed two independent methods of analyses; however, this does not completely rule out the confounding effects of cellular heterogeneity. Availability of better cell type reference sets developed by fractionation of cell types in infant cord tissue, in a population-specific manner, will alleviate this limitation in future. In spite of the lack of comprehensive reference sets, an important observation is that we did not find association between the estimated cellular proportions and birth weight for the majority of the study individuals investigated (Chinese and Indian, 75% of sample size), thus reducing the possible impact of residual confounding due to cellular heterogeneity. Second, we acknowledge that in investigating genetic influences on birth weight, our study was not designed to have sufficient power for a genome-wide association study. Indeed, such a study performed on child anthropometric outcomes from birth to 48 months of age (data not shown) revealed no single locus significant at the commonly used genome-wide significance threshold (*P* = 5 × 10^–8^). However, the absence of any single loci achieving the conventional genome-wide significance at 5 × 10^–8^ was more likely to be due to a lack of statistical power than caused by a lack of genetic influences on birth weight. Therefore, we used a genetic risk profiling approach and genetic variants reported by the GIANT consortium to form a single composite measure/score of genetic risk, and used this risk score to investigate genetic influences on birth weight. Third, the GUSTO cohort study was primarily designed to obtain extensive prenatal environment measures at mid-pregnancy. Consequently, we were unable to examine trimester-specific effects on the growing fetus. Since late pregnancy weight gain has been linked to suboptimal metabolic outcomes in offspring, we analysed maternal weight measures in late pregnancy derived from medical records (36–41 weeks, N = 803 of 987 subjects). As gestational weight gain from pre-pregnancy to mid-pregnancy already showed a significant association with birth weight, we restricted the late pregnancy analysis to the weight gain between mid-pregnancy and 36–41 weeks. Unlike the gestational weight gain up to mid-pregnancy, the additional weight gain during late pregnancy did not associate with infant birth weight (*P* = 0.12). It is unclear whether the absence of significance is an indication of trimester-specific effects or a result of low statistical power due to the reduced sample size. Future studies require detailed pre-pregnancy and trimester-specific information to reflect better on the temporal influences of prenatal environment on the growing fetus. Lastly, while we have longitudinal measures of anthropometry, we do not have longitudinal measures of methylation in early childhood and during fetal development, which would be important for determining causality and directionality of the effects. For example, to investigate if DNA methylation mediates effects of the prenatal environment on offspring adiposity one would need to first establish the temporality/directionality of the effects, i.e. whether (1) increased child adiposity leads to the alterations in DNA methylation, or (2) DNA methylation changes lead to increased child adiposity. DNA methylation is a possible mediator in the latter scenario but not the former. Moreover, examining further how DNA methylation levels at these loci change with age, body size, adiposity during childhood and environmental exposures during childhood will allow for better evaluation of these candidate loci for stratification and obesity prevention strategies. A comparison of methylation measurements collected in utero, at birth and in early childhood, across different tissue types, is an important area of investigation for future studies.

Childhood obesity has both immediate and long-term effects on the health and well-being of an individual. Children who are obese are more likely to become obese adults [64–66]. In the Bogalusa Heart study [65], childhood levels of both BMI and triceps skinfolds were associated with adult BMI and adiposity. The magnitudes of these associations increased with childhood age, but were evident from as early as 2 years of age. Overweight children (age 2– 5 years) with BMI ≥ 95th percentile had more than four times the risk of becoming overweight adults compared with children < 50th percentile. Childhood obesity is also linked with several adversities and co-morbidities in the life-course [67]. It can lead to type 2 diabetes, cardiovascular risk and increased incidence of metabolic syndrome in youth and adults. It is also associated with earlier pubertal maturation in girls, and early maturing girls tend to have higher BMIs and body fat at the time of menarche [68, 69]. Co-morbidities developed during the life-course in obese children include bone and joint problems, as well as social and psychological issues such as stigmatisation and poor self-esteem [70, 71]. A deeper understanding on how different factors contribute to adiposity, especially early in life, could be useful in troubleshooting the obesity epidemic.

## Conclusions

Developmental pathways to adiposity begin before birth and are influenced by genetic, epigenetic and prenatal environment factors. These pathways may have lasting effects on offspring size, adiposity and metabolic trajectory, and have utility in identifying individuals who are susceptible to obesity and metabolic disease later in life.

## Additional files

Additional file 1: Supplementary tables and figures. (PDF 2610 kb)
Additional file 2: Compressed folder containing a tab-delimited file with methylation values for 987 samples, 174,211 CpGs. (GZ 560574 kb)
Additional file 3: Information on using methylation data in Additional file 2. (PDF 42 kb)

## Abbreviations

DOHaD: Developmental origins of health and disease
EWAS: Epigenome-wide association studies
FDR: False discovery rate
GA: Gestational age
GUSTO: Growing Up in Singapore Towards Healthy Outcomes
GWG: Gestational weight gain
LD: Linkage disequilibrium
MUFA: Monounsaturated fatty acids
PC: Phosphatidylcholine
PUFA: Polyunsaturated fatty acids
ppBMI: Pre-pregnancy BMI
SD: Standard deviation
PRS: Polygenic risk score
T2DM: Type 2 diabetes mellitus
