# Supplementary material for: Developmental pathways to adiposity begin before birth and are influenced by genotype, prenatal environment and epigenome
Source: BMC Med. 2017 Mar 7;15:50. doi: 10.1186/s12916-017-0800-1 (PMC5340003; doi:10.1186/s12916-017-0800-1)

Developmental pathways to adiposity begin before birth and are influenced by genotype, prenatal environment and epigenome

Supplementary Material

This document contains hyperlinks on
(1) contents page (to navigate to each individual section) and
(2) bottom left corner of each page (to return to contents page).

Contents

Supplementary A: BW $\sim$ E **2**

Supplementary B: BW $\sim$ G, G = Polygenic risk score (PRS) **15**

Supplementary C: BW $\sim$ M **18**

Supplementary D: M $\sim$ E **24**

Supplementary E: M $\sim$ G **26**

Supplementary F: Y $\sim$ M, Y = outcomes in early childhood **31**

Supplementary Table A1: **Number and percentage of offspring with age- and sex-specific BMI Z-score $2 \leq Z < 3$ and ≥ 3 at each time point**. Age- and sex-specific BMI Z-score was calculated using WHO child growth charts.

Time point	Total N	N ($2 \leq Z < 3$)	% ($2 \leq Z < 3$)	N ($Z \geq 3$)	% ($Z \geq 3$)
Delivery	959	6	0.63	0	0
3 months	904	23	2.5	1	0.11
6 months	864	21	2.4	3	0.35
9 months	829	13	1.0	1	0.12
12 months	845	17	2.0	0	0
15 months	843	20	2.4	3	0.36
18 months	687	13	1.9	3	0.44
24 months	718	12	1.7	6	0.84
36 months	817	26	3.2	14	1.7
48 months	716	35	4.9	14	2.0

Supplementary Table A2: **Number and percentage of mothers who were underweight/normal/overweight/obese before pregnancy.** Maternal pre-pregnancy BMI were categorized into 4 categories. For 3rd column, WHO BMI categories were used (underweight: <18.5 kg/m^2, normal: 18.5-25 kg/m^2, overweight: 25-30 kg/m^2, obese: ≥30 kg/m^2). For 4th column, WHO Asian BMI categories were used (underweight: <18.5 kg/m^2, normal: 18.5-23.0 kg/m^2, overweight: 23.0-27.5 kg/m^2, obese: ≥ 27.5 kg/m^2). Note that the categorization is provided for information only, the analyses modeled pre-pregnancy BMI as a continuous variable.

	WHO general categorization	WHO asian categorization
Underweight	106 (12%)	106 (12%)
Normal range	579 (64%)	468 (52%)
Overweight	156 (17%)	203 (22%)
Obese	65 (7%)	129 (14%)
Total	906 (100%)	906 (100%)

Supplementary Table A3: **Prenatal environment influences on birth weight - Univariate association between birth weight and all prenatal environment variables examined**, adjusted for infant sex, ethnicity and gestational age. Regression coefficients (Est), 95% confidence intervals (CI) and p-values (P) are reported as percentage change in birth weight for two standard deviations increase in continuous prenatal environment variables, or for comparing the two categories of binary prenatal environment variables.

	Est	95% CI	P
Pre-pregnancy BMI	4.6	(2.9, 6.2)	2.5e-08
Gestational weight gain	3.6	(2.1, 5.2)	4.1e-06
Maternal Height	2.6	(1.2, 4.1)	0.00044
Fasting glucose	5.5	(4.0, 7.0)	1.2e-12
2hr post-glucose	3.1	(1.6, 4.6)	7.2e-05
n-6 PUFA	2.2	(0.60, 3.8)	0.0066
MUFA	-3.1	(-4.6, -1.5)	0.00019
Parity	1.4	(-0.052, 2.9)	0.059
Maternal age	1.8	(0.14, 3.5)	0.034
Smoking before pregnancy	-2.8	(-5.0, -0.63)	0.012
Smoking during pregnancy	-6.5	(-11, -2.2)	0.0038
n-3 PUFA	0.16	(-1.4, 1.7)	0.84
SFA	-0.078	(-1.6, 1.5)	0.92
EPDS score	0.44	(-1, 1.9)	0.56
STAI State score	0.19	(-1.3, 1.7)	0.80
STAI Trait score	-0.21	(-1.6, 1.2)	0.78
Caloric intake 3day food diary	-1.1	(-3.0, 0.86)	0.27
Caloric intake 24hr recall	0.94	(-0.52, 2.4)	0.21
Plasma Vitamin D	-1.6	(-3.9, 0.78)	0.19
Plasma Folate	-0.037	(-2.6, 2.6)	0.98
Plasma Vitamin B12	-1.5	(-3.1, 0.062)	0.060
Plasma Vitamin B6	0.22	(-1.9, 2.4)	0.84
Plasma Iron	-1.3	(-5.4, 2.9)	0.53
Plasma Zinc	0.021	(-5.2, 5.5)	0.99
Plasma Magnesium	-1.9	(-4.3, 0.54)	0.13
IVF birth	2.2	(-0.65, 5.1)	0.13
Maternal education	0.97	(-0.61, 2.6)	0.23
Maternal working	-0.61	(-2.2, 0.97)	0.45
Alcohol before pregnancy	-1.2	(-2.7, 0.43)	0.15
Alcohol during pregnancy	-2.2	(-7.2, 3.1)	0.40

Supplementary Table A4: **Prenatal environment influences on birth weight - Multivariate association between birth weight and prenatal environment variables**, adjusted for infant sex, ethnicity and gestational age. Regression coefficients (Est), 95% confidence intervals (CI) and p-values (P) are reported as percentage change in birth weight for two standard deviations increase in continuous prenatal environment variables, or for comparing the two categories of binary prenatal environment variables.

	Est	95% CI	P
Pre-pregnancy BMI	4.2	(2.3, 6.0)	8.6e-06
Gestational weight gain	4.5	(2.8, 6.3)	1.8e-07
Maternal Height	1.2	(-0.37, 2.8)	0.13
Fasting glucose	5.2	(3.5, 6.9)	2.2e-09
2hr post-glucose	2.9	(1.2, 4.6)	0.00084
n-6 PUFA	1.5	(-0.17, 3.2)	0.08
MUFA	-2.6	(-4.2, -1.0)	0.0015
Parity	1.3	(-0.35, 3.0)	0.12
Maternal age	0.93	(-0.97, 2.9)	0.34
Smoking before pregnancy	-3.2	(-5.6, -0.75)	0.011
Smoking during pregnancy	-5.5	(-10, -0.75)	0.024

Supplementary Table A5: **Prenatal environment and genetic influences on birth weight - Multivariate association between birth weight and prenatal environment variables and polygenic risk score (PRS)**, adjusted for infant sex, ethnicity and gestational age. Regression coefficients (Est), 95% confidence intervals (CI) and p-values (P) are reported as percentage change in birth weight for two standard deviations increase in continuous prenatal environment variables/PRS, or for comparing the two categories of binary prenatal environment variables.

	Est	95% CI	P
ppBMI	4.0	(2.2, 5.9)	1.5e-05
GWG	4.7	(2.9, 6.4)	8.3e-08
Height	1.1	(-0.44, 2.8)	0.16
Glucose (F)	5.1	(3.4, 6.9)	2.5e-09
Glucose (2h)	2.9	(1.2, 4.6)	0.00076
n-6 PUFA	1.4	(-0.21, 3.1)	0.087
MUFA	-2.7	(-4.3, -1.1)	0.0012
Parity	1.4	(-0.25, 3.1)	0.098
Age	0.90	(-1.0, 2.8)	0.36
Smoking (B)	-3.3	(-5.6, -0.82)	0.0091
Smoking (D)	-5.6	(-10, -0.89)	0.020
PRS	2.0	(0.44, 3.6)	0.012

Supplementary Table A6: **Prenatal environment influences on birth weight was not polygenic risk score (PRS)-dependent**, adjusted for infant sex, ethnicity and gestational age (and other prenatal environment variables). P-values (P) are for interaction term between each prenatal environment variable and PRS (with log-transformed birth weight as outcome), adjusted for the main effects of all prenatal environment variables, PRS, infant sex, ethnicity and gestational age.

	EnvxPRS (P)
ppBMI	0.56
GWG	0.99
Height	0.80
Glucose (F)	0.59
Glucose (2h)	0.35
n-6 PUFA	0.15
MUFA	0.81
Parity	0.53
Age	0.63
Smoking (B)	0.57
Smoking (D)	0.11

Supplementary Figure A1: **Study inclusion/exclusion criteria.**

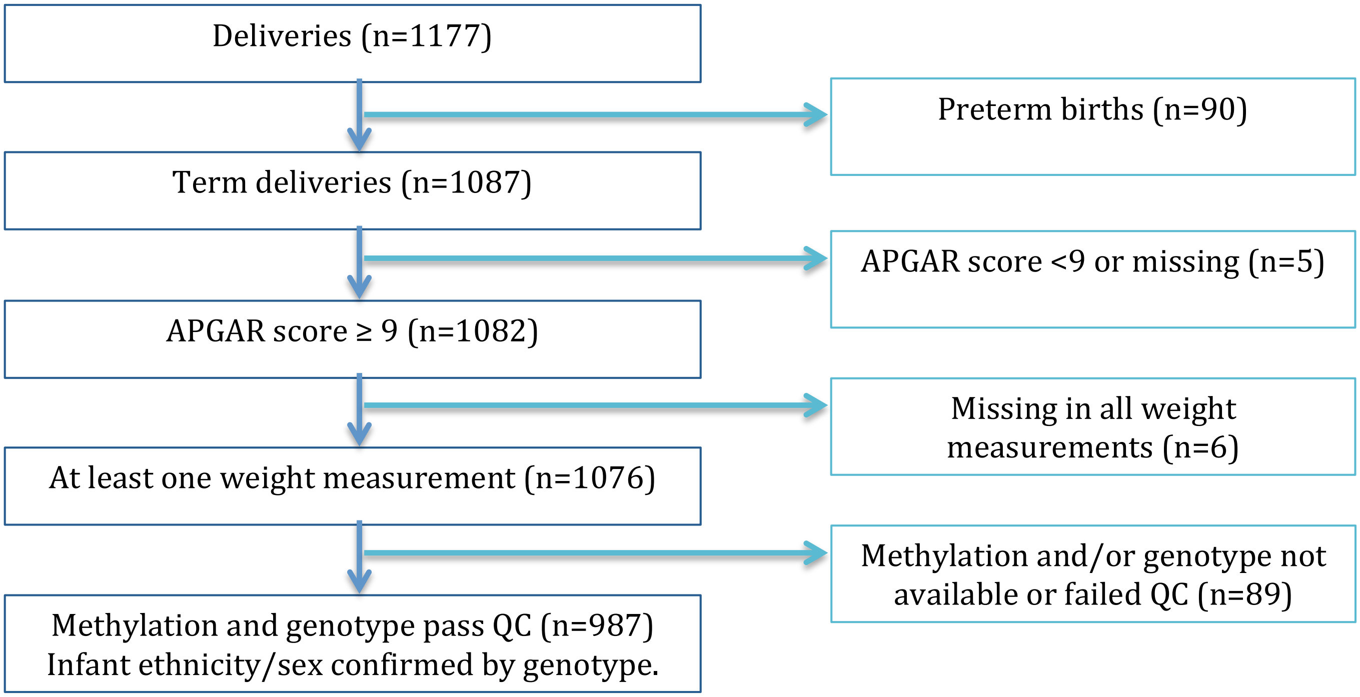

Supplementary Figure A2: **Genotype filtering criteria.**

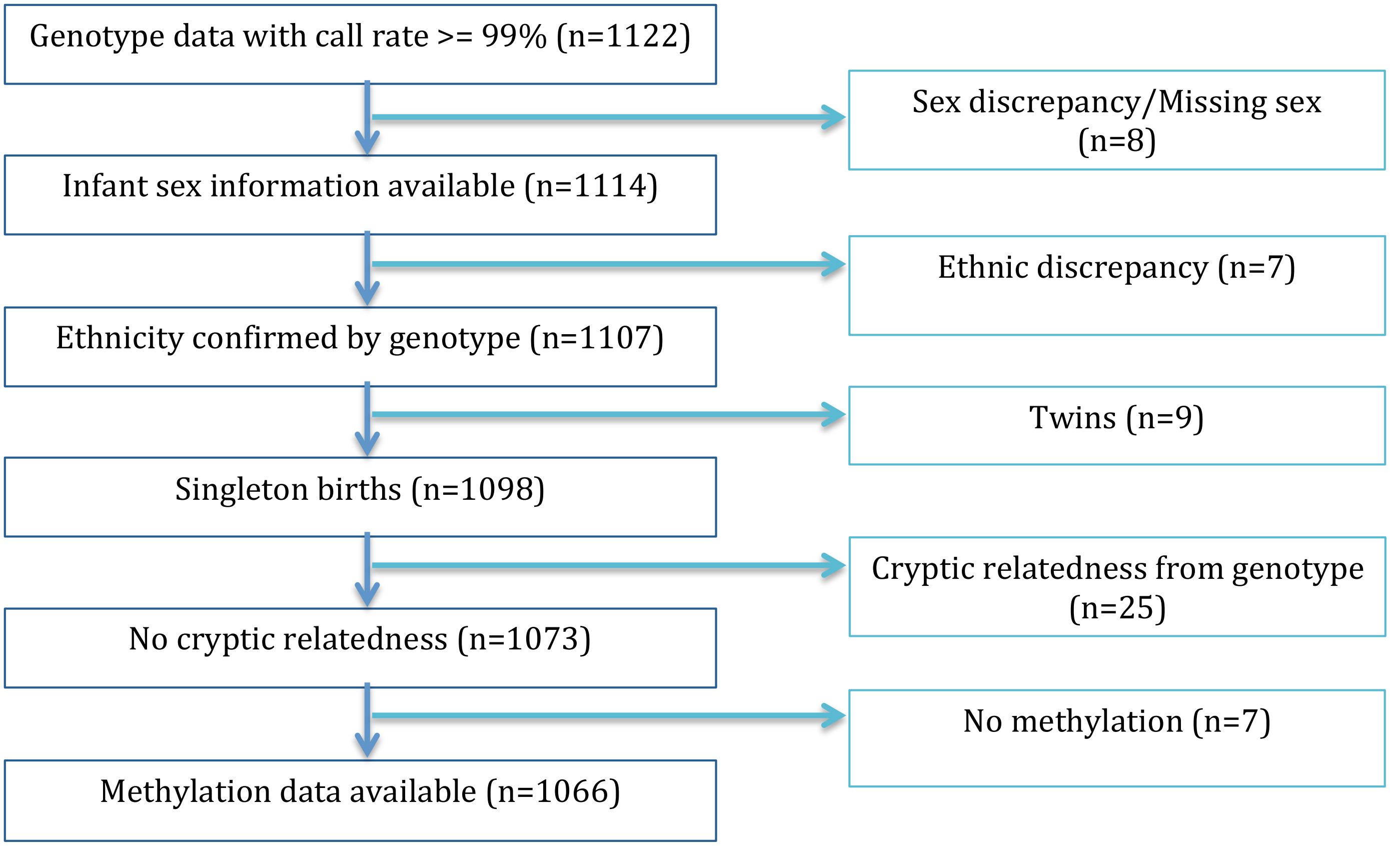

Supplementary Figure A3: **Methylation filtering criteria.**

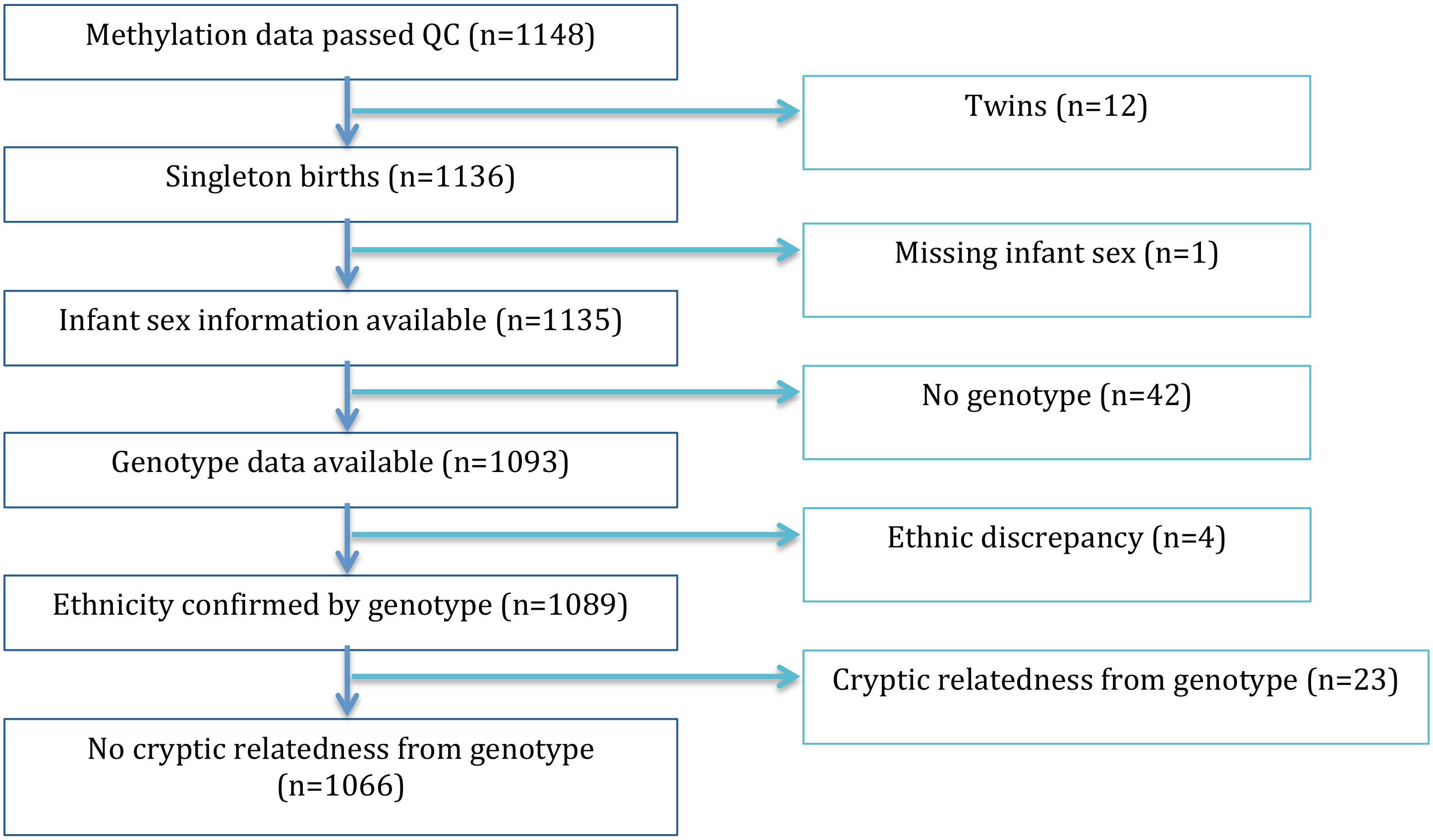

Supplementary Figure A4: **Overall analysis framework.** The overall analysis framework (bottom panel) in relation to the developmental origins of health and disease (DOHaD) hypothesis (top panel). According to the DOHaD hypothesis, the predisposition to adulthood diseases is primed *in utero* by specific prenatal environments and the mechanistic underpinnings of this phenomenon includes alterations in the epigenome. In the bottom panel, each analysis (i) to (v) is further elaborated in Methods - Statistical analysis sections (i) to (v). Directions of arrow indicate possible temporality of effects (arrows do not imply causation). Environment, methylome, phenotype (weight/adiposity/metabolic traits) can change with time and are thus represented separately at each time point. Genotype (SNPs) is largely static and thus represented as a single entity.

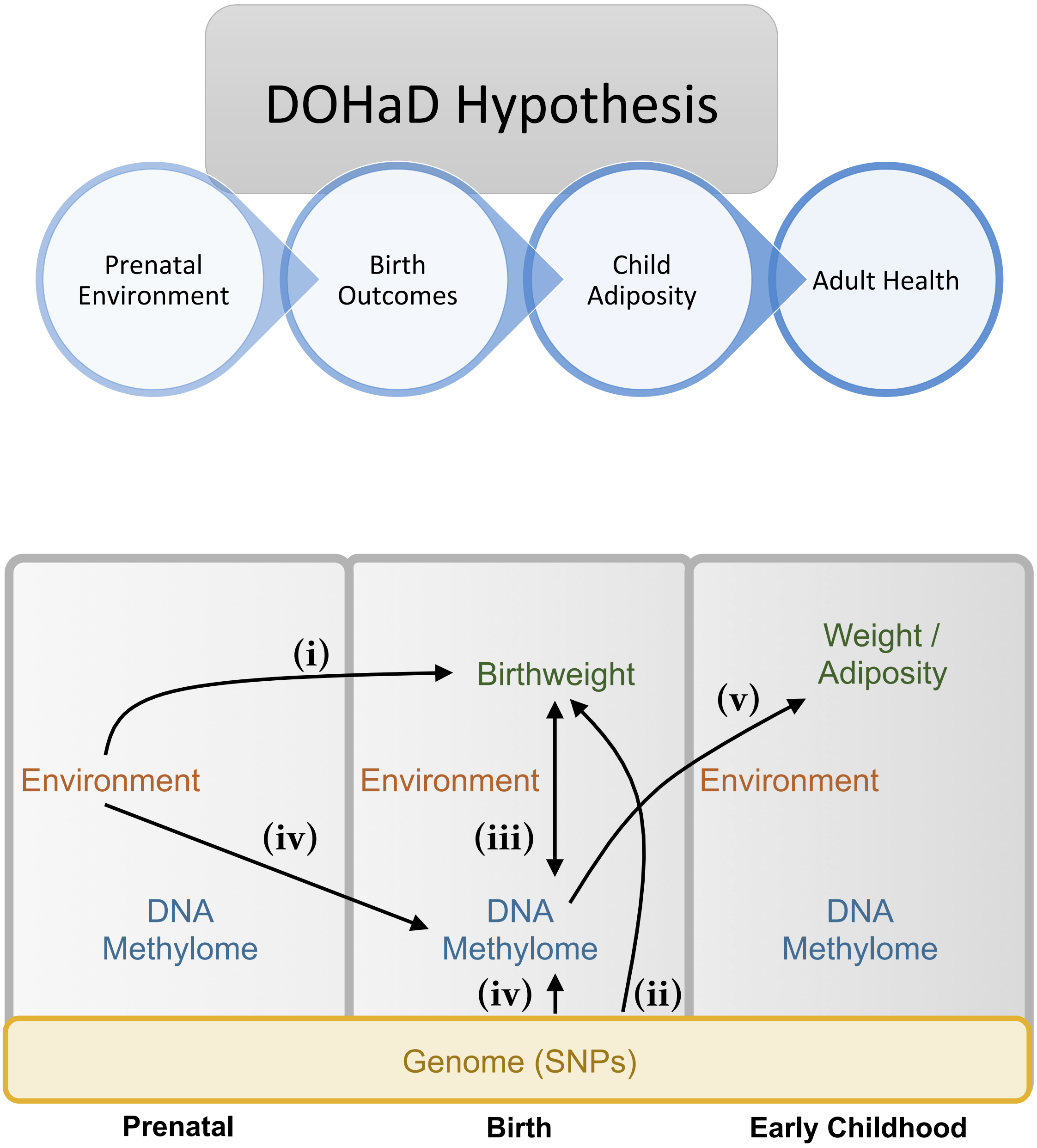

Supplementary Figure A5: **Prenatal environment influences on child weight from birth to 48 months.** Univariate association between child weight at each time point with significantly associated prenatal environment variables, adjusted for infant sex, ethnicity and gestational age. Point estimates (height of bars) and 95% confidence intervals (top and bottom whiskers) show percentage change in child weight, for two standard deviations increase in continuous prenatal environment variable, or for comparing the two categories of binary prenatal environment variables.

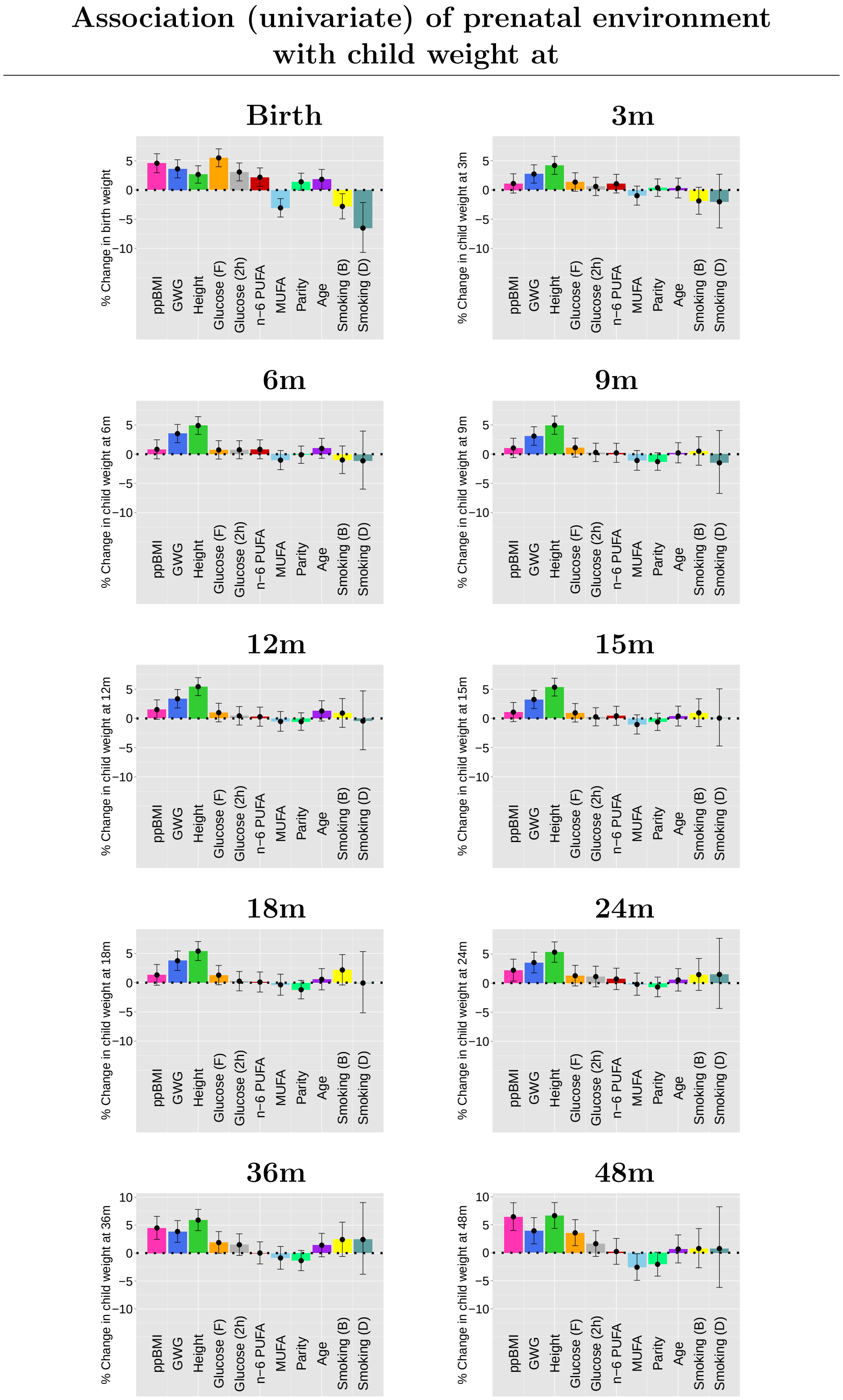

Supplementary Figure A6: **Prenatal environment influences on child BMI from birth to 48 months.** Univariate association between child BMI at each time point with significantly associated prenatal environment variables, adjusted for infant sex, ethnicity and gestational age. Point estimates (height of bars) and 95% confidence intervals (top and bottom whiskers) show percentage change in child BMI, for two standard deviations increase in continuous prenatal environment variable, or for comparing the two categories of binary prenatal environment variables.

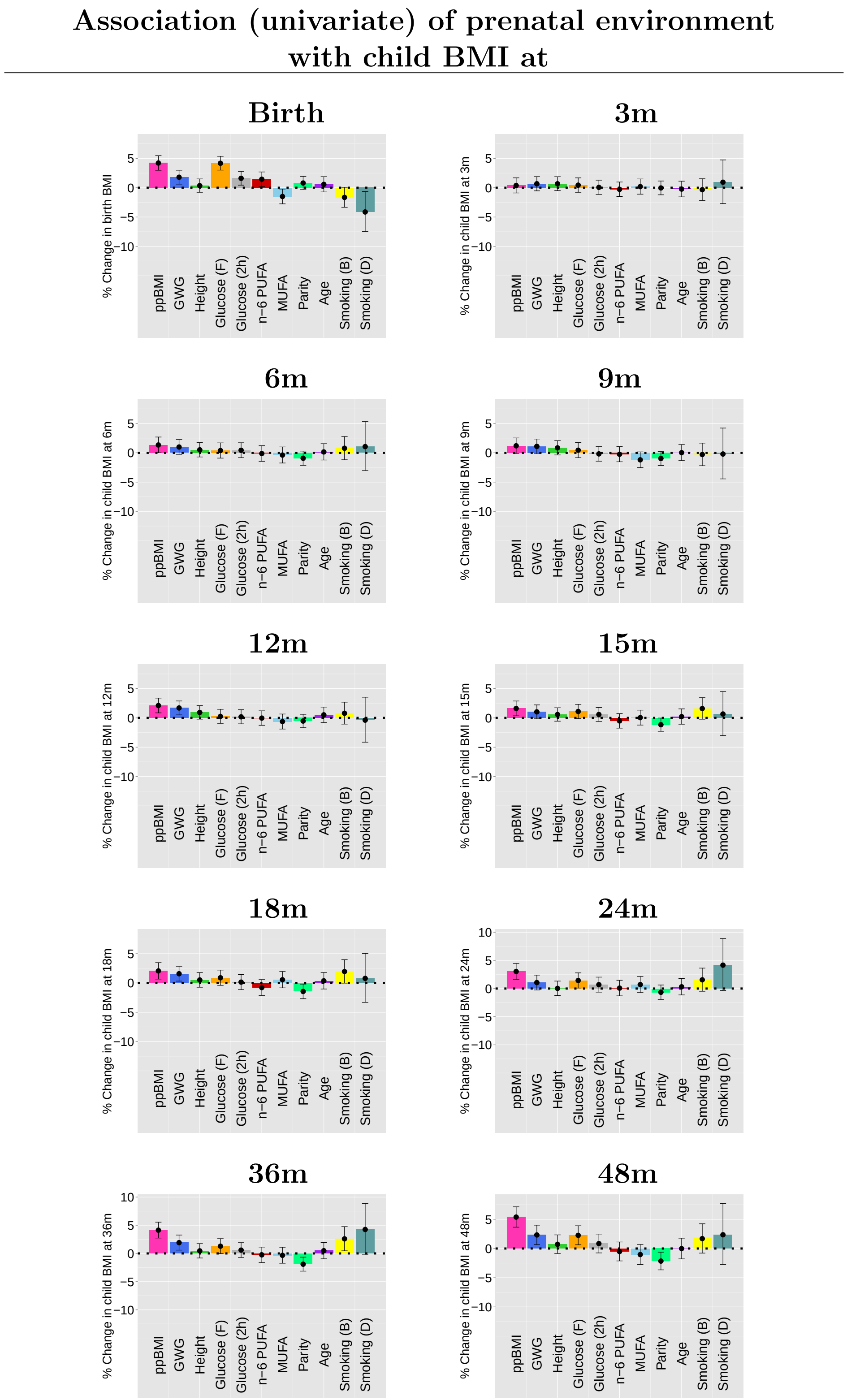

Supplementary Figure A7: **Prenatal environment influences on child weight from birth to 48 months.** Multivariate association between child weight at each time point with significantly associated prenatal environment variables, adjusted for infant sex, ethnicity, gestational age and for each other. Point estimates (height of bars) and 95% confidence intervals (top and bottom whiskers) show percentage change in child weight, for two standard deviations increase in continuous prenatal environment variable, or for comparing the two categories of binary prenatal environment variables.

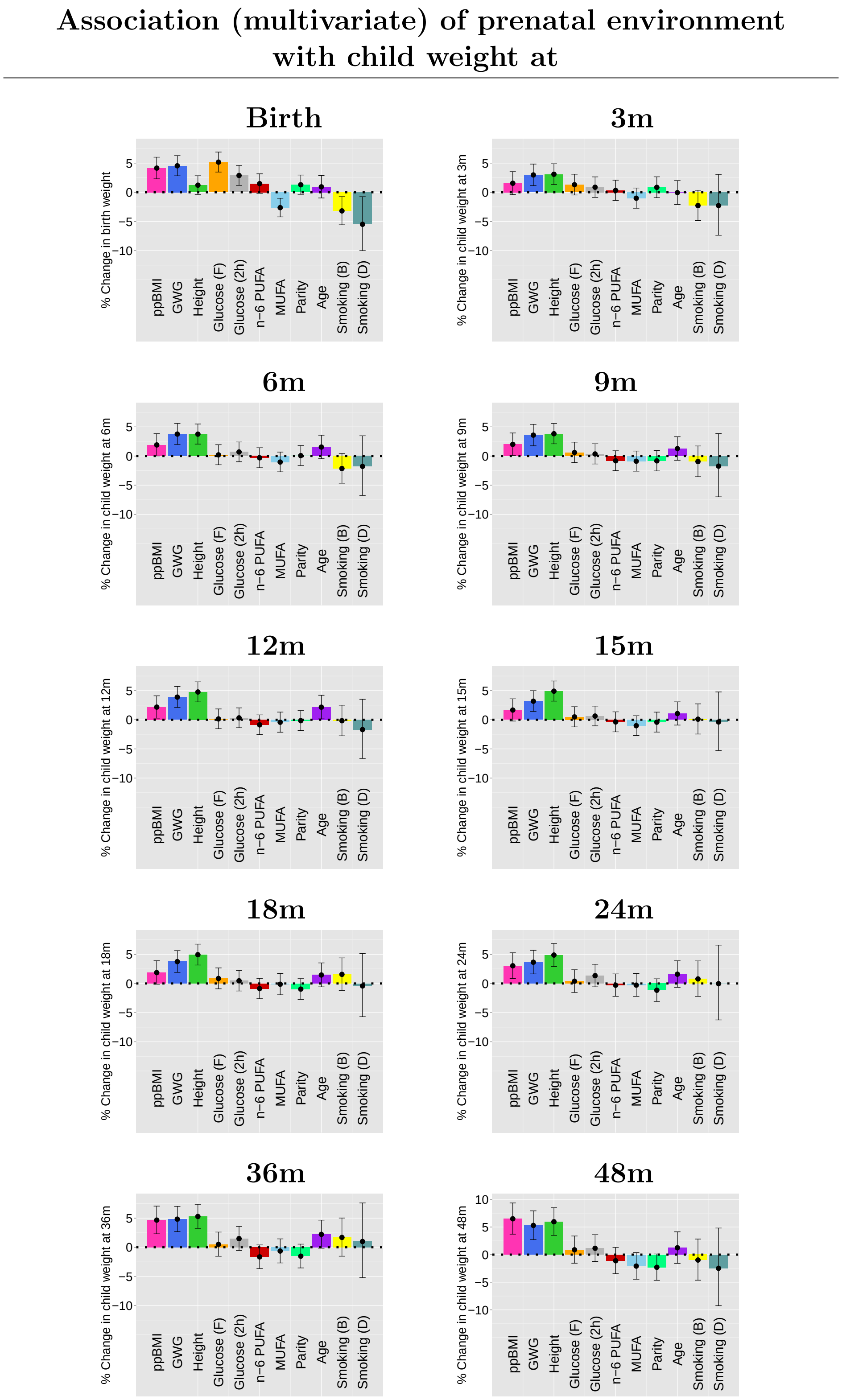

Supplementary Figure A8: **Prenatal environment influences on child BMI from birth to 48 months.** Multivariate association between child BMI at each time point with significantly associated prenatal environment variables, adjusted for infant sex, ethnicity, gestational age and for each other. Point estimates (height of bars) and 95% confidence intervals (top and bottom whiskers) show percentage change in child BMI, for two standard deviations increase in continuous prenatal environment variable, or for comparing the two categories of binary prenatal environment variables.

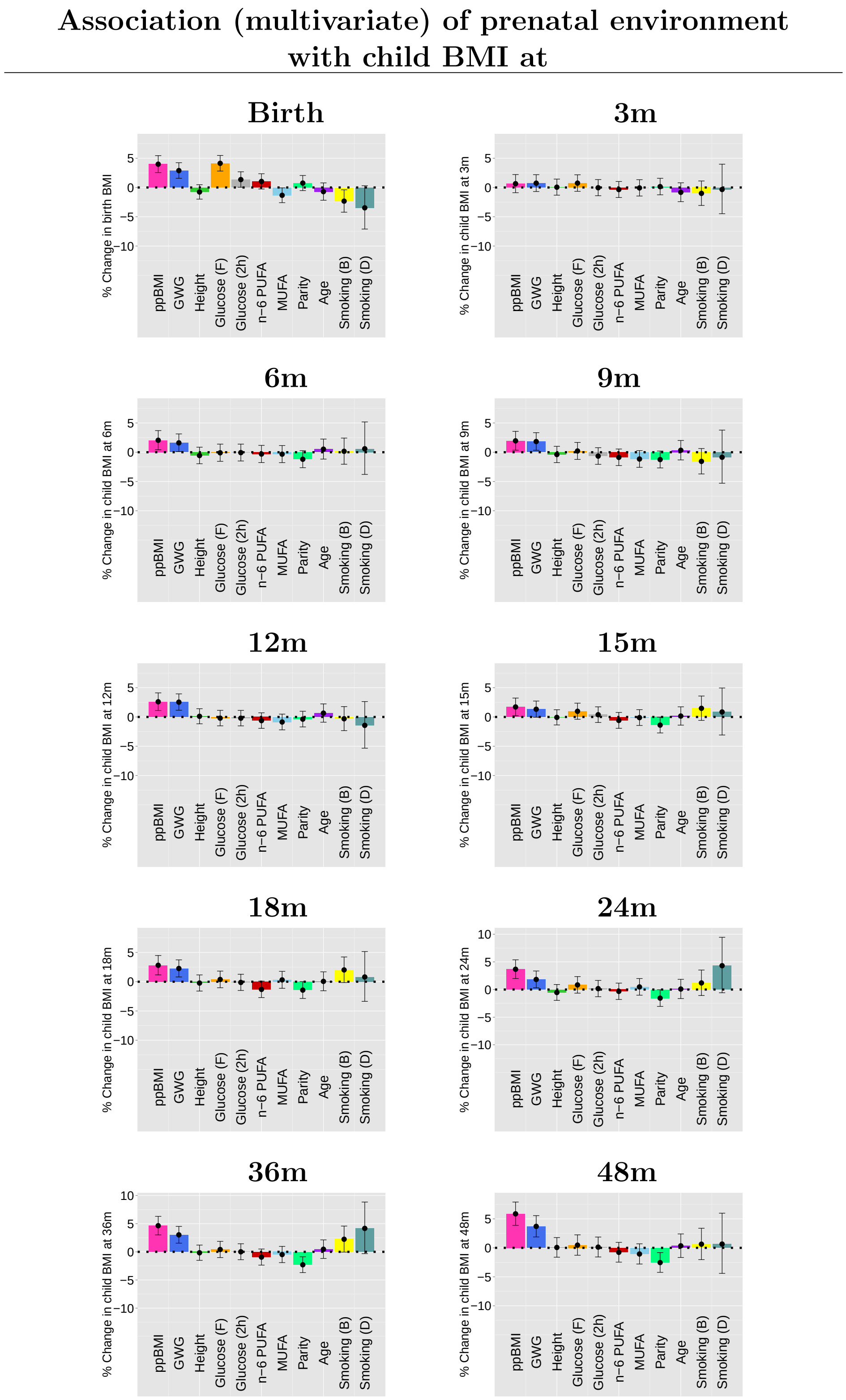

Supplementary Figure A9: **Prenatal environment and genetic influences on birth weight.** Multivariate association between birth weight, significantly associated prenatal environment variables, and polygenic risk score (PRS), adjusted for infant sex, ethnicity, gestational age and for each other. Point estimates (height of bars) and 95% confidence intervals (top and bottom whiskers) show percentage change in birth weight, for two standard deviations increase in continuous prenatal environment variable/PRS, or for comparing the two categories of binary prenatal environment variables.

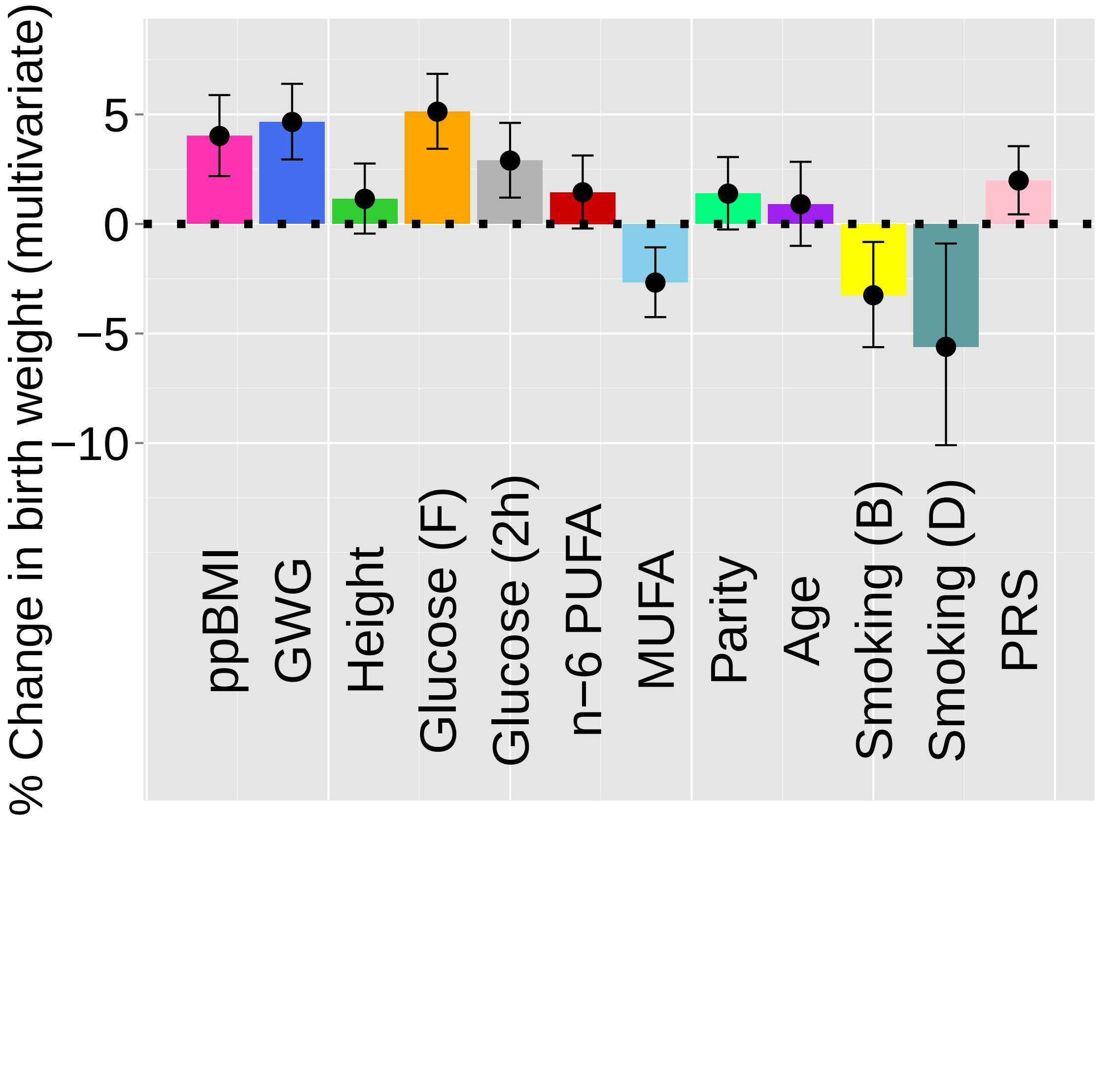

Supplementary Table B1: **Associations of child anthropometry at different time points with best-fit polygenic risk score (PRS).** Best-fit PRS for Chinese, Malay and Indian ethnic groups used clumping p-value thresholds $p_T = 0.5, 0.1$ and 10^{-4}, respectively. PRS was standardized to mean zero and unit variance within each ethnic group. Regression coefficients (Est), 95% confidence intervals (CI) and p-values (P) are reported as percentage change in child outcome, for two standard deviations increase in PRS, adjusted for child sex, gestational age and ethnicity. Analysis was done by linear regression of log-transformed child anthropometric outcome at each time point against PRS, adjusted for child sex, gestational age and ethnicity. These results are also shown on left panel of Supplementary Figure B1. MalayxPRS (P) and IndianxPRS (P) give p-values assessing whether the associations of child anthropometry with PRS depend on ethnicity. Analysis was done by linear regression of log-transformed child anthropometric outcome at each time point against interactions terms between PRS and ethnicity, adjusted for main effects of PRS, ethnicity, child sex and gestational age.

Child weight

	Est	95% CI	P	MalayxPRS (P)	IndianxPRS (P)
Birth	1.6	(0.21, 3.1)	0.024	0.47	0.9
3 months	2.3	(0.78, 3.8)	0.0028	0.93	0.11
6 months	2.5	(1.1, 4.1)	0.00079	0.43	0.56
9 months	1.9	(0.44, 3.5)	0.011	0.02	0.62
12 months	2.6	(1.1, 4.1)	0.00072	0.38	0.38
15 months	2.6	(1.1, 4.1)	0.00056	0.26	0.27
18 months	2.3	(0.74, 4)	0.004	0.31	0.085
24 months	3.8	(2.1, 5.5)	1.4e-05	0.89	0.41
36 months	4.2	(2.4, 6.1)	5.9e-06	0.81	0.27
48 months	5.7	(3.4, 7.9)	4.7e-07	0.096	0.11

Child BMI

	Est	95% CI	P	MalayxPRS (P)	IndianxPRS (P)
Birth	1.5	(0.41, 2.7)	0.0072	0.79	0.9
3 months	1.8	(0.67, 3)	0.0021	0.53	0.41
6 months	2	(0.77, 3.2)	0.0014	0.52	0.37
9 months	1.6	(0.38, 2.8)	0.0097	0.008	0.52
12 months	1.8	(0.68, 3)	0.0017	0.73	0.31
15 months	2	(0.88, 3.2)	0.00051	0.2	0.19
18 months	2.2	(0.93, 3.4)	0.00057	0.11	0.03
24 months	3	(1.7, 4.3)	4.3e-06	0.42	0.17
36 months	3.2	(1.9, 4.5)	6.5e-07	0.49	0.22
48 months	4.6	(3, 6.2)	7.3e-09	0.092	0.12

Child subscapular skinfold

	Est	95% CI	P	MalayxPRS (P)	IndianxPRS (P)
Birth	0.38	(-2.4, 3.2)	0.79	0.85	0.83
18 months	1.5	(-1.5, 4.6)	0.33	0.64	0.00013
24 months	4.9	(1.7, 8.1)	0.0022	0.25	0.017
36 months	3.2	(-0.27, 6.8)	0.071	0.45	0.032
48 months	9.5	(4.8, 14)	6.1e-05	0.037	0.016

Child triceps skinfold

	Est	95% CI	P	MalayxPRS (P)	IndianxPRS (P)
Birth	0.44	(-2.3, 3.3)	0.76	0.65	0.46
18 months	0.98	(-1.9, 4)	0.51	0.77	0.02
24 months	1.1	(-1.8, 4.2)	0.46	0.62	0.02
36 months	0.84	(-2.4, 4.2)	0.62	0.62	0.09
48 months	4.8	(0.82, 9)	0.018	0.014	0.0069

Child subscapular:triceps ratio (STR)

	Est	95% CI	P	MalayxPRS (P)	IndianxPRS (P)
Birth	-0.058	(-2.3, 2.2)	0.96	0.74	0.52
18 months	0.55	(-2.2, 3.4)	0.7	0.96	0.099
24 months	3.7	(1, 6.4)	0.0063	0.31	0.86
36 months	2.4	(-0.34, 5.3)	0.085	0.78	0.71
48 months	4.8	(2, 7.6)	0.00065	0.77	0.92

Child length/height

	Est	95% CI	P	MalayxPRS (P)	IndianxPRS (P)
Birth	0.059	(-0.38, 0.5)	0.79	0.4	0.96
3 months	0.21	(-0.25, 0.67)	0.38	0.35	0.13
6 months	0.32	(-0.18, 0.82)	0.22	0.044	0.62
9 months	0.17	(-0.32, 0.66)	0.51	0.77	0.95
12 months	0.4	(-0.11, 0.91)	0.12	0.37	0.87
15 months	0.26	(-0.24, 0.77)	0.3	0.76	0.89
18 months	0.22	(-0.34, 0.79)	0.44	0.27	0.62
24 months	0.56	(-0.0089, 1.1)	0.054	0.64	0.37
36 months	0.55	(0.029, 1.1)	0.038	0.81	0.91
48 months	0.51	(-0.065, 1.1)	0.082	0.39	0.35

Supplementary Figure B1: **Associations of child anthropometry at different time points with best-fit polygenic risk score (PRS).** Best-fit PRS for Chinese, Malay and Indian ethnic groups used clumping p-value thresholds $p_T = 0.5, 0.1$ and 10^{-4}, respectively. PRS was standardized to mean zero and unit variance within each ethnic group. Left panel shows point estimates (height of bars) and 95% confidence intervals (top and bottom whiskers), for percentage change in child outcome, for two standard deviations increase in PRS, adjusted for child sex, gestational age and ethnicity. Analysis was done by linear regression of log-transformed child anthropometric outcome at each time point against PRS, adjusted for child sex, gestational age and ethnicity. Right panel shows scatterplot of standardized (mean zero and unit variance) log-transformed child outcome (vertical axis) against PRS (horizontal axis).

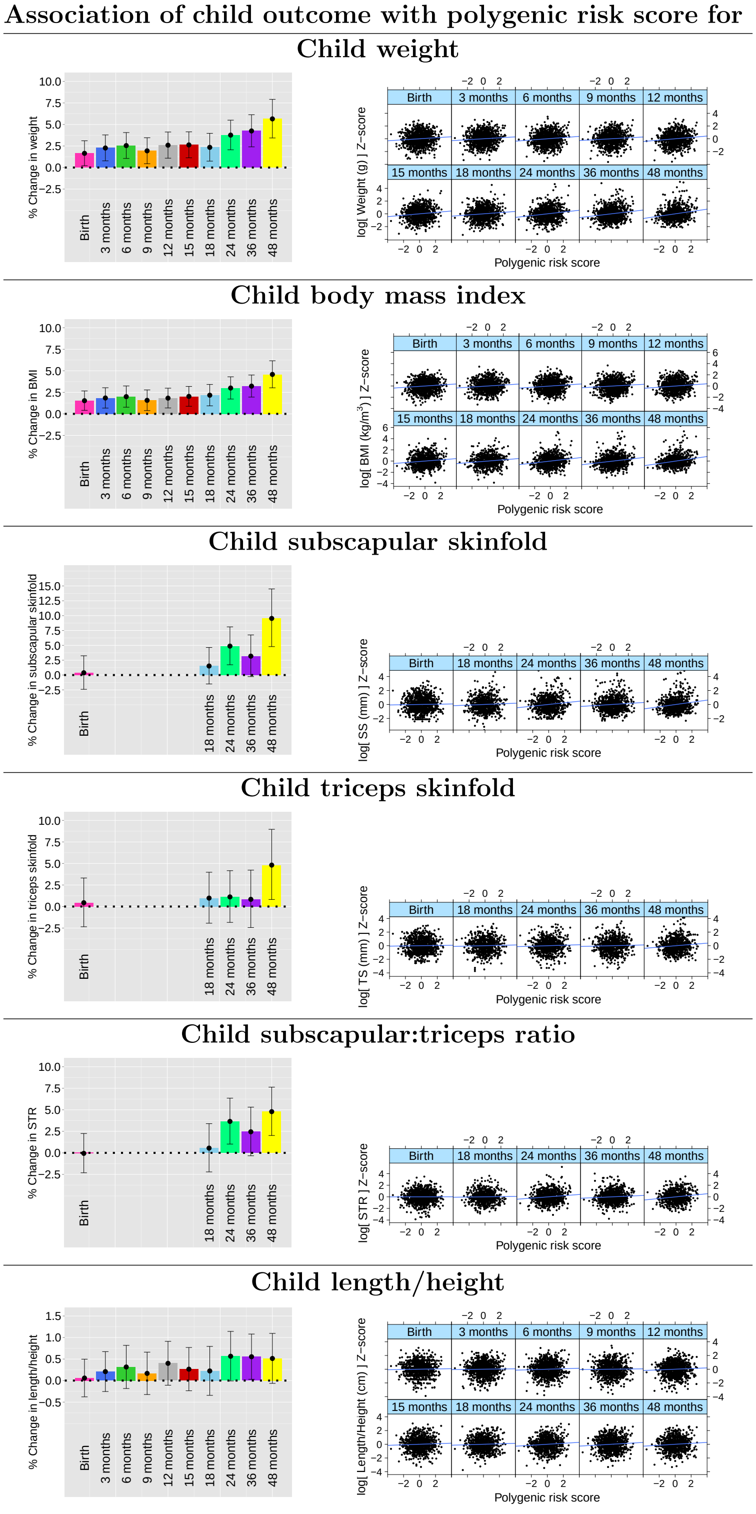

Supplementary Figure B2: **Associations of child anthropometry at different time points with best-fit polygenic risk score (PRS), adjusted for birth anthropometric outcome.** Best-fit PRS for Chinese, Malay and Indian ethnic groups used clumping p-value thresholds $p_T = 0.5, 0.1$ and 10^{-4}, respectively. PRS was standardized to mean zero and unit variance within each ethnic group. Point estimates (height of bars) and 95% confidence intervals (top and bottom whiskers), are for percentage change in child outcome, for two standard deviations increase in PRS, adjusted for child sex, gestational age, ethnicity and the corresponding anthropometric outcome at birth. Analysis was done by linear regression of log-transformed child anthropometric outcome at each time point against PRS, adjusted for child sex, gestational age, ethnicity and corresponding (log-transformed) anthropometric outcome at birth. Results without adjustment for child anthropometric outcome at birth are reported in Supplementary Figure B1.

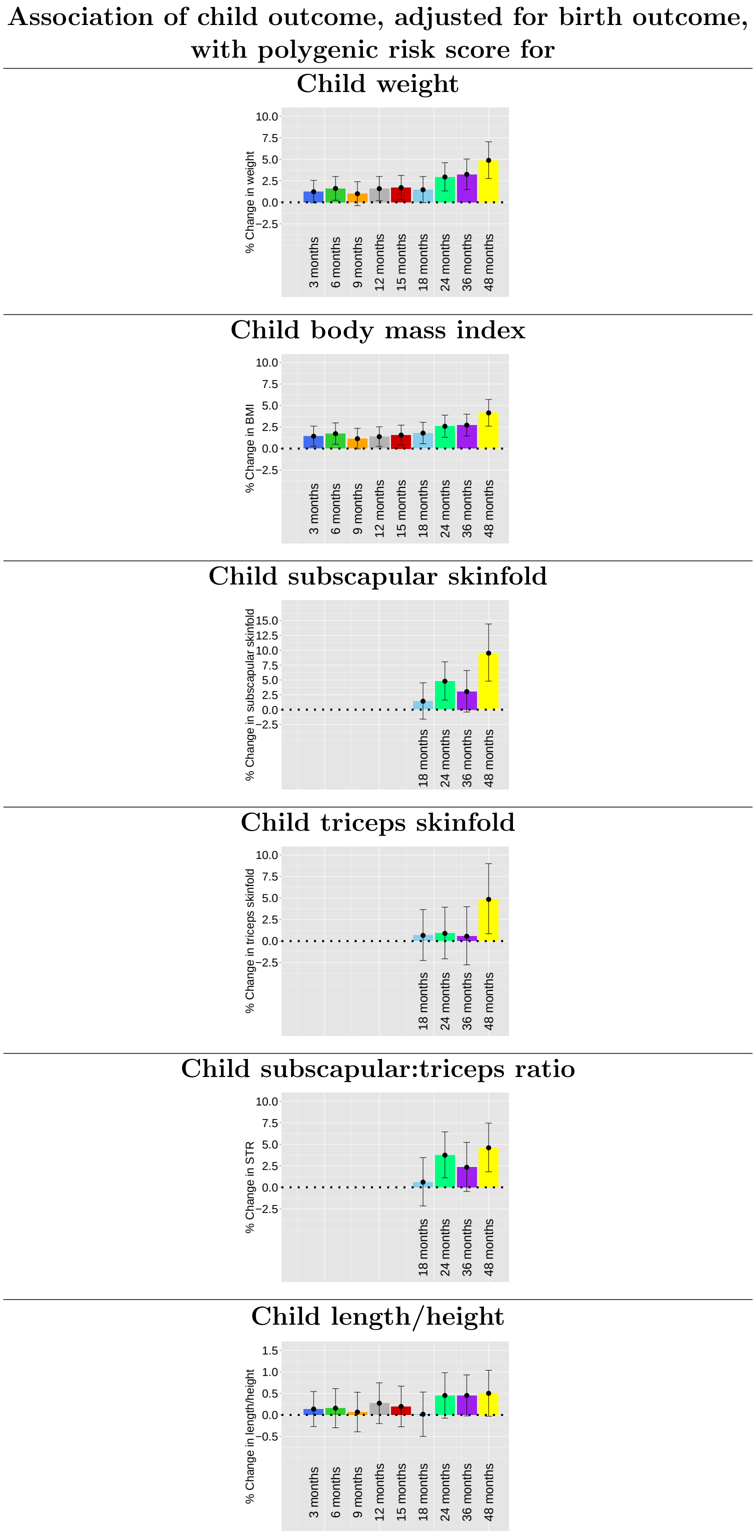

Supplementary Figure C1: **CpGs which showed inter-individual variation were more likely to be located in open seas and intronic/intergenic regions.** Functional genomic distribution (left panel) and CpG content distribution (right panel) of CpGs which showed inter-individual variation (top panel) and CpGs which did not show inter-individual variation (bottom panel). A total of 374,650 CpGs passed quality control, however only 174,211 CpGs showed inter-individual variation (top panel).

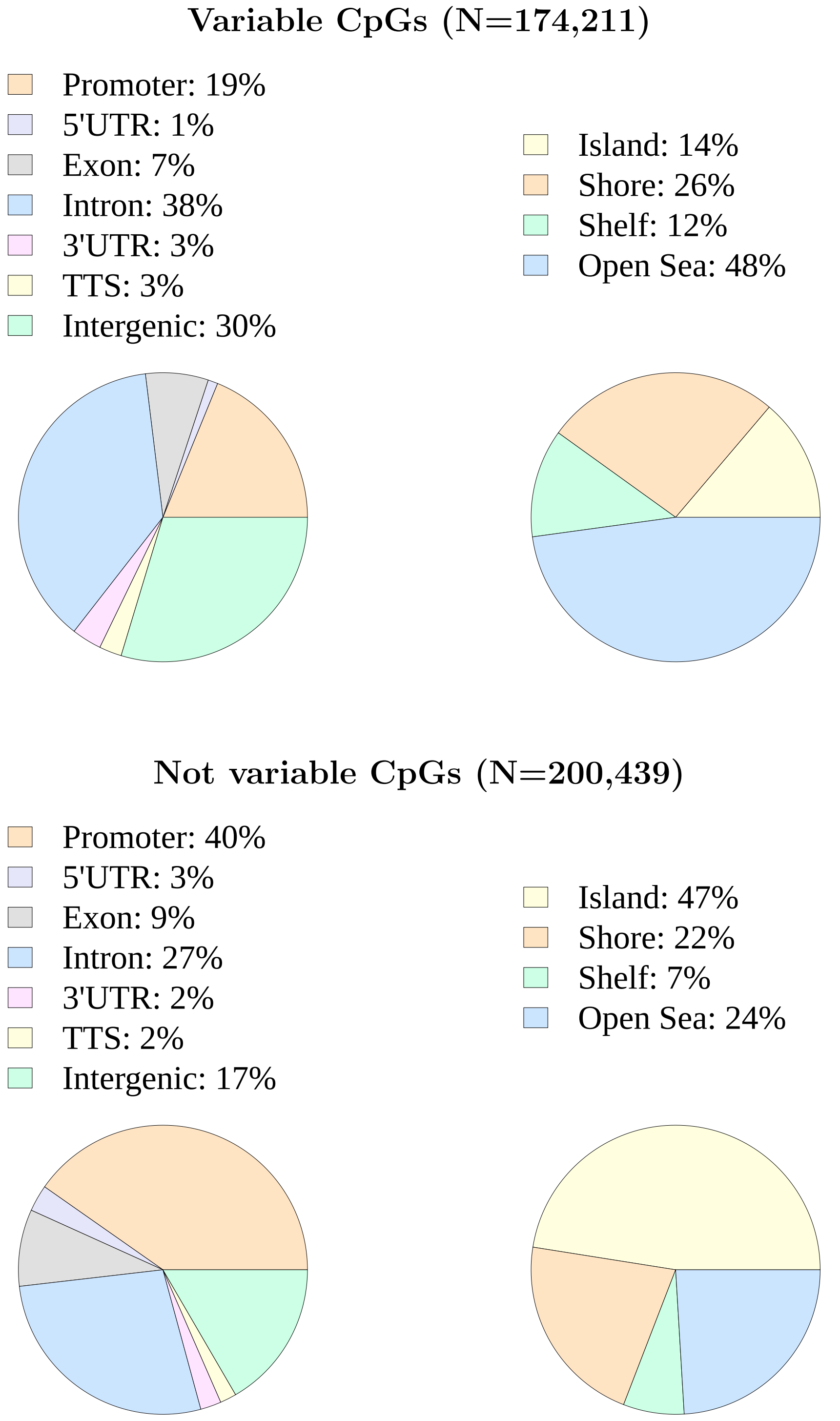

Supplementary Figure C2: **Manhattan plot of p-values for associations between methylation and birth weight.** Analysis was done by linear regression of log-transformed birth weight against methylation at each CpG site, adjusted for child sex, gestational age, ethnicity, cellular proportions and interactions between ethnicity and cellular proportions. Association p-values are represented in genomic order by chromosome and position on the horizontal axis and $-\log_{10}$p-value on the vertical axis. Horizontal red line indicates false discovery rate threshold at 0.05.

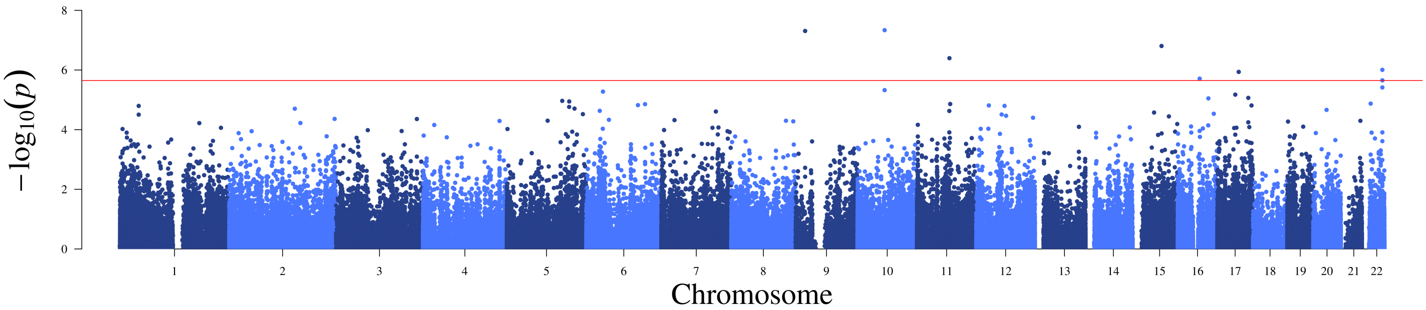

Supplementary Figure C3: **Scatterplots of top 8 CpGs associated with birth weight.** The top 8 CpGs were obtained from regressing log-transformed birth weight against percent methylation, adjusted for child sex, gestational age, ethnicity, cellular proportions and interactions between ethnicity and cellular proportions (Table 3 in main text). Vertical axis gives birth weight and horizontal axis gives standardized methylation values (outliers truncated).

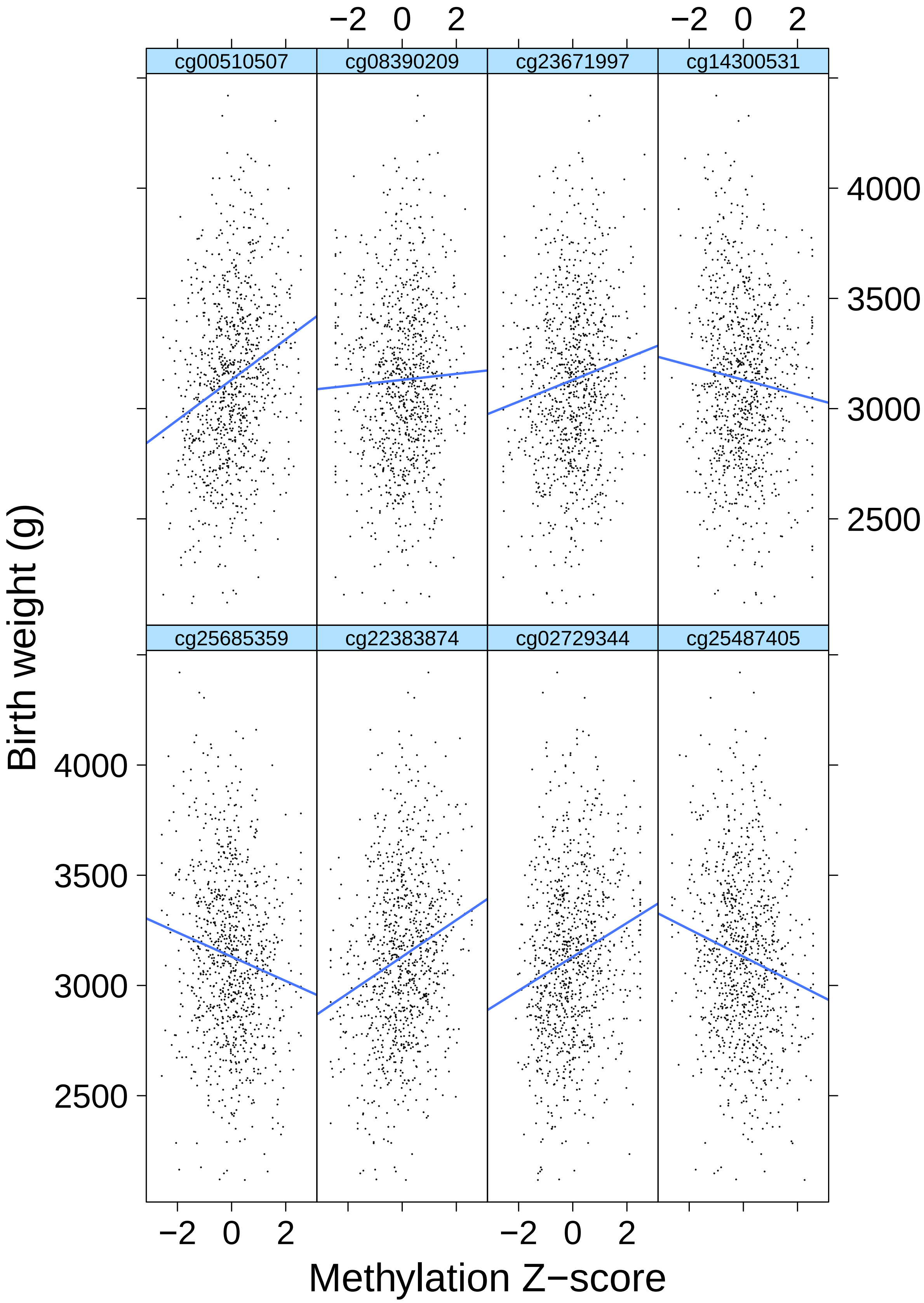

Supplementary Table C1: **Methylome - Top 8 CpGs associated with birth weight, adjusted for cellular heterogeneity using reference-free method (surrogate variable analysis).** The top 8 CpGs (Table 3 in main text) were obtained from regressing log-transformed birth weight against percent methylation and adjusted for estimated cellular proportions which were calculated using a referenced-based method (Houseman, 2012). This table reports associations for these 8 CpGs, but adjusted for cellular heterogeneity using a reference-free method (surrogate variable analysis). Regression coefficients (Est), 95% confidence intervals (CI) and p-values (P) are reported as percentage change in birth weight for 10% increase in percent methylation. Interquartilerange (IQR), chromosome (CHR) and position (POS) of CpG are also shown. Analysis was done by linear regression of log-transformed birth weight against methylation at each CpG site, adjusted for child sex, gestational age, ethnicity and surrogate variables (Leek, 2007).

CpG	CHR	POS	IQR	Est	95% CI	P	Gene
cg00510507	10	61900413	8.4	5.8	(4.3, 7.3)	6.5e-08	*ANK3*
cg08390209	9	22005563	6.6	8.6	(6.1, 11)	1.1e-06	*CDKN2B*
cg23671997	15	65677753	4.6	13	(9.1, 16)	3.0e-07	*IGDCC4*
cg14300531	11	73969506	9.6	-4.3	(-5.5, -3.0)	3.7e-06	*P4HA3*
cg25685359	22	46473721	8.8	-5.2	(-6.5, -3.9)	1.2e-07	*MIRLET7BHG*
cg22383874	17	48670670	4.8	13	(9.5, 16)	1.5e-08	*CACNA1G*
cg02729344	16	49888237	6.6	8.4	(5.9, 11)	1.8e-06	*ZNF423*
cg25487405	22	46473039	5.5	-7.7	(-9.7, -5.6)	4.8e-07	*MIRLET7BHG*

Supplementary Table C2: **Methylome - associations of top 8 CpGs with birth weight, were not ethnic-dependent.** The top 8 CpGs (Table 3 in main text) were obtained from regressing log-transformed birth weight against percent methylation, adjusted for child sex, gestational age, ethnicity, cellular proportions and interactions between ethnicity and cellular proportions. This table gives p-values for assessing whether these associations were ethnic-dependent. Analysis was done by regressing log-transformed birth weight against interaction terms between methylation and ethnicity, adjusted for main effects of methylation, main effects of ethnicity, child sex, gestational age, cellular proportions and interactions between ethnicity and cellular proportions.

CpG	MalayxCpG (P)	IndianxCpG (P)
cg00510507	0.39	0.042
cg08390209	0.64	0.80
cg23671997	0.87	0.93
cg14300531	0.28	0.42
cg25685359	0.97	0.49
cg22383874	0.76	0.84
cg02729344	0.78	0.84
cg25487405	0.96	0.48

Supplementary Table C3: **Methylome - associations of top 8 CpGs with birth weight, were not (child) sex-dependent.** The top 8 CpGs (Table 3 in main text) were obtained from regressing log-transformed birth weight against percent methylation, adjusted for child sex, gestational age, ethnicity, cellular proportions and interactions between ethnicity and cellular proportions. This table gives p-values for assessing whether these associations were (child) sex-dependent. Analysis was done by regressing log-transformed birth weight against interaction terms between methylation and child sex, adjusted for main effects of methylation, main effects of child sex, gestational age, ethnicity, cellular proportions and interactions between ethnicity and cellular proportions.

CpG	CpGxInfantSex (P)
cg00510507	0.40
cg08390209	0.37
cg23671997	0.96
cg14300531	0.39
cg25685359	0.70
cg22383874	0.81
cg02729344	0.82
cg25487405	0.38

Supplementary Table C4: **Methylome - CpGs previously reported to be associated with birth weight.** Thirty-four CpGs that were previously reported to be associated with birth weight passed quality control and showed inter-individual variation in our study (last column). For these CpGs, regression coefficients (Est), 95% confidence intervals (CI) and p-values (P) are reported as percentage change in birth weight for 10% increase in percent methylation. Interquartilerange (IQR), chromosome (CHR) and position (POS) of CpG are also shown. Analysis was done by linear regression of log-transformed birth weight against methylation at each CpG site, adjusted for child sex, gestational age, ethnicity, cellular proportions and interactions between ethnicity and cellular proportions. To adjust for multiple testing across the 34 previously reported CpGs, a CpG would be considered replicated in our study (statistically significant) if p-value $< \frac{0.05}{34} \approx 1 \times 10^{-3}$. Only 1 CpG had p-value $< 1 \times 10^{-3}$ (cg04521626, which mapped to *PLD2* gene). Cord blood methylation at cg04521626 was previously reported to be negatively associated with birth weight; we observed a positive association between cord tissue methylation and birth weight.

CpG	Publication	CHR	POS	IQR	Est	95% CI	P	Gene	Notes
cg25953130	Engel *et al.*, Simpkin *et al.*	10	63753550	2.7	-2.4	(-6.2, 1.7)	3.9e-01	*ARID5B*	Variable
cg20076442	Engel *et al.*, Simpkin *et al.*	8	72745197	6	0.54	(-2.4, 3.6)	7.9e-01	Intergenic	Variable
cg08005122	Engel *et al.*	11	110779637	4.4	-2.8	(-5, -0.6)	6.3e-02	Intergenic	Variable
cg02863179	Engel *et al.*	10	63779053	6.8	0.036	(-1.3, 1.4)	9.7e-01	*ARID5B*	Variable
cg25124943	Engel *et al.*	10	4117248	7.2	-2.6	(-4.1, -1.2)	7.7e-03	*LOC101927964*	Variable
cg00049440	Engel *et al.*	9	73026643	6.7	-1.4	(-2.8, 0.081)	1.7e-01	*KLF9*	Variable
cg02194129	Engel *et al.*	14	104171313	4.6	0.58	(-1.9, 3.1)	7.3e-01	*XRCC3*	Variable
cg17836177	Engel *et al.*	8	22735111	4.7	2.5	(0.52, 4.6)	6.6e-02	*PEBP4*	Variable
cg12798040	Engel *et al.*	14	104171840	4.4	1.7	(-0.8, 4.3)	3.2e-01	*XRCC3*	Variable
cg00605777	Engel *et al.*	2	97533635	4.3	0.35	(-2.1, 2.8)	8.4e-01	*SEMA4C*	Variable
cg14172849	Engel *et al.*	14	104171259	4.7	0.0045	(-2.7, 2.8)	1	*XRCC3*	Variable
cg23127323	Engel *et al.*	4	1196179	7	0.86	(-0.48, 2.2)	3.5e-01	*SPON2*	Variable
cg25162533	Engel *et al.*	17	81032067	4.4	0.44	(-2.3, 3.3)	8.2e-01	Intergenic	Variable
cg23369670	Engel *et al.*	14	104171944	4.8	3.2	(1, 5.4)	3.1e-02	*XRCC3*	Variable
cg17714703	Engel *et al.*	19	4912221	3	-1.7	(-5.1, 1.8)	4.7e-01	*UHRF1*	Variable
cg08420923	Engel *et al.*	16	89436447	5	0.44	(-3.5, 4.5)	8.7e-01	*ANKRD11*	Variable
cg23237276	Engel *et al.*	14	101827449	4.3	1.8	(-1.2, 4.9)	3.8e-01	Intergenic	Variable
cg05993265	Engel *et al.*	4	2933802	4.4	1.4	(-1, 3.8)	4.1e-01	*MFSD10*	Variable
cg24693803	Engel *et al.*								Failed QC
cg18566515	Haworth *et al.*	10	123356236	7.9	0.061	(-1.3, 1.4)	9.5e-01	*FGFR2*	Variable
cg27258878	Sharp *et al.*								Failed QC
cg06019229	Sharp *et al.*								Not Variable
cg19351954	Sharp *et al.*	7	156259207	5.5	-1.2	(-2.9, 0.58)	3.3e-01	Intergenic	Variable
cg01963623	Sharp *et al.*	22	18632433	6	-1.4	(-3, 0.23)	2.1e-01	*USP18*	Variable
cg04521626	Simpkin *et al.*	17	4714200	6.4	3.7	(2.2, 5.2)	3.0e-04	*PLD2*	Variable
cg14097568	Simpkin *et al.*	1	94792942	6.8	-1.6	(-2.9, -0.15)	1.0e-01	Intergenic	Variable
cg17133774	Simpkin *et al.*	1	6198667	5	-0.78	(-2.8, 1.3)	5.8e-01	*CHD5*	Variable
cg00654448	Simpkin *et al.*	8	142365315	4.9	0.59	(-1.7, 2.9)	7.1e-01	Intergenic	Variable
cg00442282	Simpkin *et al.*								Not Variable
cg13696490	Simpkin *et al.*	3	151488006	5.9	-0.35	(-2, 1.3)	7.6e-01	*AADACL2-AS1*	Variable
cg12044213	Simpkin *et al.*	6	31124978	3.5	-2.2	(-4.6, 0.31)	2.0e-01	*CCHCR1*	Variable
cg08817867	Simpkin *et al.*	17	19656554	6.4	-0.52	(-2.3, 1.3)	6.7e-01	Intergenic	Variable
cg00382138	Simpkin *et al.*								Failed QC
cg06870470	Simpkin *et al.*	19	11315767	6.6	-1.3	(-2.9, 0.34)	2.5e-01	*DOCK6*	Variable
cg25557739	Simpkin *et al.*								Not Variable
cg24324628	Simpkin *et al.*	6	138866882	11	0.56	(-0.42, 1.6)	4.0e-01	*NHSL1*	Variable
cg15783941	Simpkin *et al.*	19	13132221	2.7	-0.005	(-1.4, 1.4)	1	*NFIX*	Variable
cg14597739	Simpkin *et al.*								Failed QC
cg22962123	Simpkin *et al.*	7	27153605	13	2.3	(0.56, 4.1)	5.2e-02	*HOXA3*	Variable
cg05851442	Simpkin *et al.*	7	27153212	11	0.57	(-1.2, 2.4)	6.4e-01	*HOXA3*	Variable
cg23387597	Simpkin *et al.*	10	106093778	5.8	-1.8	(-3.5, 0.025)	1.5e-01	*ITPRIP*	Variable
cg24973755	Simpkin *et al.*								Failed QC
cg16219283	Simpkin *et al.*								Not Variable
cg25799241	Simpkin *et al.*								Failed QC
cg06658067	Simpkin *et al.*								Failed QC

Supplementary Table D1: **Influence of prenatal environment on the top 8 CpGs associated with birth weight.** Second column gives the prenatal environment variable for which the association was strongest (smallest p-value) and third column gives the p-value for this association. Analysis was done by linear regression of methylation at each CpG site against each prenatal environment variable, adjusted for child sex, gestational age, ethnicity, cellular proportions and interactions between ethnicity and cellular proportions. A CpG was defined to be influenced by the prenatal environment if the most significant association between the CpG and prenatal environment attained a p-value $< 5 \times 10^{-4}$, the bonferroni threshold to maintain a family-wise Type 1 error rate of 0.05 across 8 CpGs $\times$ 11 prenatal environment variables $\sim$ 100 tests. CpGs that were influenced by the prenatal environment are indicated with a (#) beside the CpG name.

CpG	Most significant prenatal environment	Most significant P
cg00510507	Glucose (F)	0.0057
cg08390209	Glucose (F)	0.0044
cg23671997 (#)	Glucose (F)	0.00027
cg14300531	Glucose (F)	0.025
cg25685359 (#)	Smoking (B)	0.00023
cg22383874 (#)	Glucose (F)	0.00017
cg02729344	Glucose (2h)	0.053
cg25487405	Smoking (B)	0.0026

Supplementary Figure D1: **Influence of prenatal environment on methylome at birth adjusted for maternal pre-pregnancy body mass index (ppBMI).** Associations of DNA methylation at birth with prenatal environment, adjusted for ppBMI. Color in heatmap represents regression coefficients for associations between methylation and each prenatal environment variable. Each row represents a CpG and each column represents a prenatal environment variable. With increasing magnitudes, color changes from white to red (for negative coefficients) or from white to blue (for positive coefficients). Asterisks within each square represent p-values for associations between methylation and each prenatal environment variable ($P < 5 \times 10^{-8}$ is represented with 8 asterisks, $5 \times 10^{-8} \leq P < 5 \times 10^{-7}$ is represented with 7 asterisks, $\cdots$, $5 \times 10^{-3} \leq P < 5 \times 10^{-2}$ is represented with 2 asterisks, $P \geq 5 \times 10^{-2}$ is represented with a blank square). Analysis was done by linear regression of methylation at each CpG site against each prenatal environment variable, adjusted for maternal ppBMI, child sex, gestational age, ethnicity, cellular proportions and interactions between ethnicity and cellular proportions. Regression coefficients and p-values are reported as increase in percent methylation for two standard deviations increase in continuous prenatal environment variable, or for comparing the two categories of binary prenatal environment variables. Analysis without adjustment for maternal ppBMI is reported in main Figure 3A.

Association of methylation with prenatal environment, adjusted for ppBMI

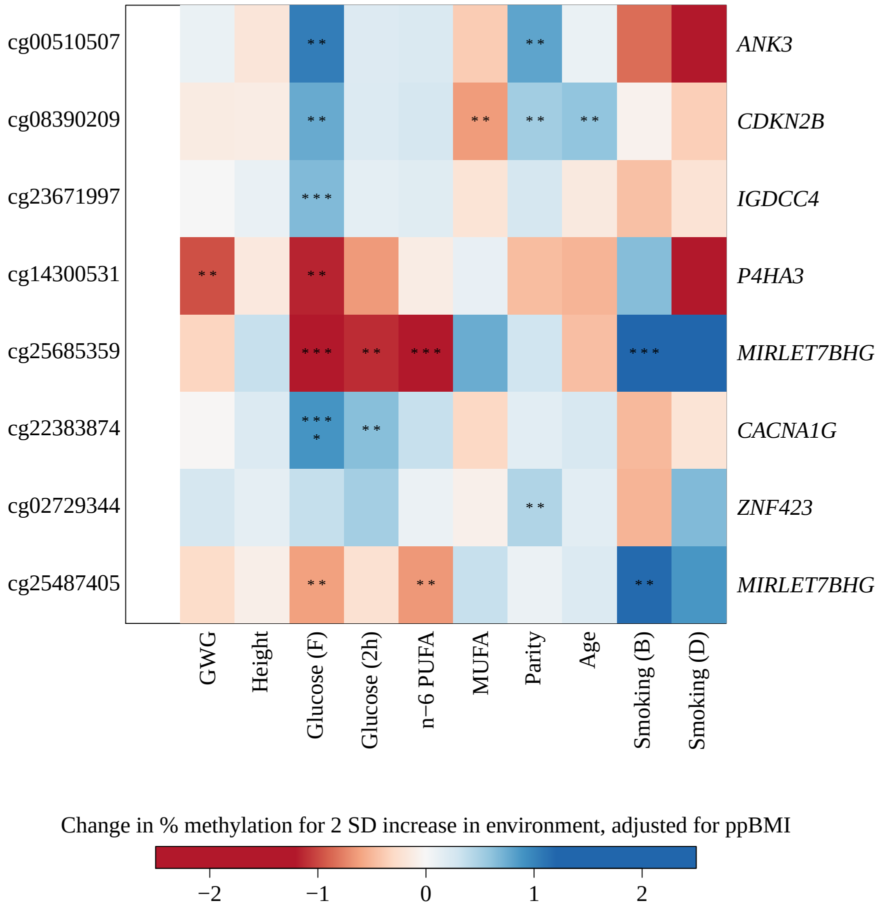

Supplementary Table E1: **Associations of top 8 CpGs with most significant *cis*-SNPs - 3 out of the 8 top CpGs associated with birth weight were associated with SNPs.** The top 8 CpGs were obtained from regressing log-transformed birth weight against percent methylation, adjusted for child sex, gestational age, ethnicity, cellular proportions and interactions between ethnicity and cellular proportions (Table 3 in main text). Each of these 8 CpGs were then regressed against genome-wide *cis*-SNPs (SNPs on the same chromosome as CpG), and the most significant *cis*-SNP (second column) for the CpG (first column) identified. The CpG-SNP associations were adjusted for child sex, gestational age, ethnicity, cellular proportions and interactions between ethnicity and cellular proportions. Third column gives distance (DIST) between CpG and SNP. Chromosome (CHR), position (POS), minor allele (A1), major allele (A2), number of individuals in each of the three genotype groups (GENO) and minor allele frequency (MAF) of SNP are also shown. Regression coefficients (Est), 95% confidence intervals (CI) and p-values (P) are reported as change in percent methylation for each additional copy of the minor allele (adjusted for child sex, gestational age, ethnicity, cellular proportions and interactions between ethnicity and cellular proportions). For SNPs where the minor homozygote genotype group had ≤ 50 individuals, the minor homozygote and heterozygote genotype groups were combined (dominant genetic model). A total of $\sim 5 \times 10^5$ CpG-SNP tests were conducted, which corresponds to testing 8 CpGs across 8,392-47,298 *cis*-SNPs for each CpG (each CpG was tested against 8,392-47,298 *cis*-SNPs depending on the chromosome of the CpG). A CpG was defined to be influenced by the genome (or showed association with the genome) if the most significant association between the CpG and genome-wide *cis*-SNPs attained a p-value $< 1 \times 10^{-7}$, the bonferroni threshold to maintain a family-wise Type 1 error rate of 0.05 across $\sim 5 \times 10^5$ tests. CpGs that were influenced by the genome (or showed association with the genome) are indicated with an asterisk beside the CpG name.

CpG	SNP	DIST	CHR	POS	Est	95% CI	P	A1	A2	GENO	MAF
cg00510507	rs10821689	-10712	10	61911125	-2.5	(-3.5, -1.4)	3.5e-06	T	C	4/116/867	0.063
cg08390209	exm-rs2383207	-110396	9	22115959	-0.84	(-1.2, -0.5)	1.8e-06	A	G	125/422/440	0.34
cg23671997	rs1495186	26686297	15	38991456	-0.94	(-1.4, -0.48)	8.0e-05	A	G	9/168/810	0.094
cg14300531 (*)	rs612040	-168045	11	74137551	-1.9	(-2.4, -1.4)	2.1e-12	A	C	194/481/312	0.44
cg25685359	rs718476	-1774999	22	48248720	-1.1	(-1.6, -0.51)	0.00019	G	A	231/503/253	0.49
cg22383874	rs9894590	36964484	17	11706186	-0.61	(-0.89, -0.32)	3.6e-05	T	C	149/455/383	0.38
cg02729344 (*)	rs16948031	24733	16	49863504	-1.9	(-2.2, -1.5)	4.4e-26	A	G	56/340/591	0.23
cg25487405 (*)	rs7291040	-11934	22	46484973	1.8	(1.2, 2.4)	1.1e-08	C	T	10/196/781	0.11

Supplementary Table E2: **Associations between birth weight and methylation were independent of SNPs.** The top 8 CpGs were obtained from regressing log-transformed birth weight against percent methylation, adjusted for child sex, gestational age, ethnicity, cellular proportions and interactions between ethnicity and cellular proportions (Table 3 in main text). Three out of the 8 top CpGs associated with birth weight were also associated with SNPs (Supplementary Table E1). We report additional results for these 3 CpG-SNP pairs below. For all 3 CpG-SNP pairs, the association between birth weight and methylation was similar without (model 1) and with adjustment for SNP (model 2), SNP was not associated with birth weight (model 3), and there was no evidence to suggest that the association between birth weight and methylation depended on SNP (model 4). Model 1 gives regression coefficients (Est), 95% confidence intervals (CI) and p-values (P) corresponding to percentage change in birth weight for 10% increase in percent methylation. Analysis was done by linear regression of log-transformed birth weight against methylation at each CpG site, adjusted for child sex, gestational age, ethnicity, cellular proportions and interactions between ethnicity and cellular proportions. Model 2 is similar to model 1 but adjusted additionally for SNP. Model 3 gives effect estimates for percentage change in birth weight for each additional copy of the minor allele. Analysis was done by linear regression of log-transformed birth weight against SNP, adjusted for child sex, gestational age, ethnicity, cellular proportions and interactions between ethnicity and cellular proportions. Model 4 is similar to both models 1 and 3 but included main effects of methylation and SNP and their interaction; The SNPxmethylation interaction p-value is reported. All 4 models had the same sample size (only individuals with birth weight, methylation and SNP were included).

	Model 1			Model 2			Model 3			Model 4
	$Y \sim M$			$Y \sim M$, adj SNP			$Y \sim$ SNP			$Y \sim M + \text{SNP} + \text{SNP} \times M$
CpG	Est	95% CI	P	Est	95% CI	P	Est	95% CI	P	P (SNP $\times$ M)
cg14300531	-3.9	(-5.0, -2.8)	4.0e-07	-4.1	(-5.2, -3.0)	3.7e-07	0.19	(-0.81, 1.2)	0.71	0.77
cg02729344	6.8	(4.7, 9.0)	1.9e-06	7.0	(4.7, 9.3)	5.0e-06	-0.97	(-2.1, 0.22)	0.11	0.98
cg25487405	-5.6	(-7.2, -3.9)	2.2e-06	-6.0	(-7.6, -4.3)	7.6e-07	1.3	(-0.51, 3.1)	0.16	0.44

Supplementary Table E3: **Top 8 CpGs associated with birth weight were not associated with polygenic risk score (PRS).** The top 8 CpGs (first column) were obtained from regressing log-transformed birth weight against percent methylation, adjusted for child sex, gestational age, ethnicity, cellular proportions and interactions between ethnicity and cellular proportions (Table 3 in main text). Each of these 8 CpGs were then regressed against PRS, adjusted for child sex, gestational age, ethnicity, cellular proportions and interactions between ethnicity and cellular proportions. Best-fit PRS for Chinese, Malay and Indian ethnic groups used clumping p-value thresholds $p_T = 0.5, 0.1$ and 10^{-4}, respectively. PRS was standardized to mean zero and unit variance within each ethnic group. Regression coefficients (Est), 95% confidence intervals (CI) and p-values (P) are reported as change in percent methylation for two standard deviations increase in PRS.

CpG	Est	95% CI	P
cg00510507	0.47	(-0.22, 1.2)	0.18
cg08390209	0.33	(-0.14, 0.81)	0.17
cg23671997	0.34	(-0.017, 0.70)	0.062
cg14300531	0.54	(-0.23, 1.3)	0.17
cg25685359	0.32	(-0.46, 1.1)	0.43
cg22383874	0.25	(-0.15, 0.65)	0.23
cg02729344	0.15	(-0.28, 0.59)	0.49
cg25487405	-0.11	(-0.61, 0.39)	0.66

Supplementary Table E4: **Associations between birth weight and methylation were independent of PRS.** The top 8 CpGs were obtained from regressing log-transformed birth weight against percent methylation, adjusted for child sex, gestational age, ethnicity, cellular proportions and interactions between ethnicity and cellular proportions (Table 3 in main text). For the top 8 CpGs, the association between birth weight and methylation was similar without (model 1) and with adjustment for PRS (model 2), and there was no evidence to suggest that the association between birth weight and methylation depended on PRS (model 4). Model 1 gives regression coefficients (Est), 95% confidence intervals (CI) and p-values (P) corresponding to percentage change in birth weight for 10% increase in percent methylation. Analysis was done by linear regression of log-transformed birth weight against methylation at each CpG site, adjusted for child sex, gestational age, ethnicity, cellular proportions and interactions between ethnicity and cellular proportions. Model 2 is similar to model 1 but adjusted additionally for PRS. Model 4 includes all the variables in model 2 and an additional interaction term between methylation and PRS; The PRSxmethylation interaction p-value is reported. All 3 models had the same sample size (only individuals with birth weight, methylation and PRS were included).

	Model 1			Model 2			Model 4
	$Y \sim M$			$Y \sim M$, adj PRS			$Y \sim M + \text{PRS} + \text{PRS} \times M$
CpG	Est	95% CI	P	Est	95% CI	P	P (PRS $\times M$)
cg00510507	4.9	(3.5, 6.2)	4.6e-08	4.8	(3.5, 6.1)	6.3e-08	0.21
cg08390209	7.1	(5.1, 9.0)	4.9e-08	7.0	(5.0, 8.9)	6.9e-08	0.43
cg23671997	9.2	(6.5, 12)	1.6e-07	9.0	(6.4, 12)	2.4e-07	0.29
cg14300531	-3.9	(-5.0, -2.8)	4.0e-07	-4.0	(-5.1, -2.9)	2.7e-07	0.53
cg25685359	-3.7	(-4.8, -2.6)	9.9e-07	-3.8	(-4.9, -2.7)	7.6e-07	0.98
cg22383874	7.6	(5.2, 10)	1.2e-06	7.5	(5.2, 9.9)	1.4e-06	0.34
cg02729344	6.8	(4.7, 9.0)	1.9e-06	6.8	(4.6, 8.9)	2.3e-06	0.11
cg25487405	-5.6	(-7.2, -3.9)	2.2e-06	-5.5	(-7.2, -3.8)	2.6e-06	0.69

Supplementary Table E5: **Associations of prenatal environmental variable with methylation were similar with and without adjustment for PRS.** The top 8 CpGs were obtained from regressing log-transformed birth weight against percent methylation, adjusted for child sex, gestational age, ethnicity, cellular proportions and interactions between ethnicity and cellular proportions (Table 3 in main text). Three of the top 8 CpGs also showed association with the prenatal environment ($P < 5 \times 10^{-4}$). For these 3 CpGs, we list the prenatal environmental variable showing the strongest association with it and conducted further analyses. The association of prenatal environmental variable with methylation was similar without (model 1) and with adjustment for PRS (model 2) and there was generally no evidence to suggest that the association between prenatal environmental variable and methylation depended on PRS (model 4). Model 1 gives regression coefficients (Est), 95% confidence intervals (CI) and p-values (P) corresponding to increase in percent methylation for two standard deviations increase in prenatal environmental variable (for continuous prenatal environmental variables) or for comparing two categories of prenatal environmental variable (for binary prenatal environmental variables). Analysis was done by linear regression of methylation at each CpG site against prenatal environmental variable, adjusted for child sex, gestational age, ethnicity, cellular proportions and interactions between ethnicity and cellular proportions. Model 2 is similar to model 1 but adjusted additionally for PRS. Model 4 includes all the variables in model 2 and an additional interaction term between prenatal environmental variable and PRS; The PRSxE interaction p-value is reported. All 3 models had the same sample size (for each CpG, only individuals with prenatal environmental variable, methylation and PRS were included).

		Model 1			Model 2			Model 4
		$M \sim E$			$M \sim E$, adj PRS			$M \sim \text{PRS} + E + \text{PRS} \times E$
CpG	Env	Est	95% CI	P	Est	95% CI	P	P (PRS × E)
cg23671997	Glucose (F)	0.80	(0.43, 1.2)	0.00027	0.79	(0.41, 1.2)	0.0011	0.053
cg25685359	Smoking (B)	2.4	(1.2, 3.6)	0.00023	2.4	(1.2, 3.6)	0.0033	0.42
cg22383874	Glucose (F)	0.93	(0.51, 1.4)	0.00017	0.93	(0.51, 1.3)	0.00067	0.45

Supplementary Table F1: **Methylome - Associations with BMI change in early childhood (difference between BMI Z-score at 48 months and BMI Z-score at birth).** Regression coefficients (Est), 95% confidence intervals (CI) and p-values (P) are reported as change in BMI change for 10% increase in percent methylation. Analysis was done by linear regression of BMI change (difference between BMI Z-score at 48 months and BMI Z-score at birth) against methylation at each CpG site, adjusted for child sex, gestational age, ethnicity, cellular proportions and interactions between ethnicity and cellular proportions. BMI Z-score at birth and 48 months were calculated using WHO child growth charts; BMI change was calculated as the difference between BMI Z-score at 48 months and BMI Z-score at birth).

CpG	Est	95% CI	P
cg00510507	-0.15	(-0.33, 0.030)	0.13
cg08390209	-0.059	(-0.32, 0.20)	0.68
cg23671997	-0.25	(-0.60, 0.097)	0.19
cg14300531	0.11	(-0.050, 0.28)	0.20
cg25685359	0.23	(0.071, 0.40)	0.0085
cg22383874	-0.068	(-0.38, 0.24)	0.69
cg02729344	0.0088	(-0.28, 0.29)	0.96
cg25487405	0.22	(-0.029, 0.47)	0.10

Supplementary Figure F1: **Influence of methylome at birth on subscapular skinfold in early childhood.** Associations of child subscapular skinfold at different time points with DNA methylation at birth. Color in heatmap represents regression coefficients for associations between child anthropometric outcome and methylation. Each row represents a CpG and each column represents a time point. With increasing magnitudes, color changes from white to red (for negative coefficients) or from white to grey (for positive coefficients). Asterisks within each square represent p-values for associations between child anthropometric outcome and methylation ($P < 5 \times 10^{-8}$ is represented with 8 asterisks, $5 \times 10^{-8} \leq P < 5 \times 10^{-7}$ is represented with 7 asterisks, $\cdots$, $5 \times 10^{-3} \leq P < 5 \times 10^{-2}$ is represented with 2 asterisks, $P \geq 5 \times 10^{-2}$ is represented with a blank square). Analysis was done by linear regression of log-transformed child anthropometric outcome at each time point against methylation at each CpG site, adjusted for child sex, gestational age, ethnicity, cellular proportions and interactions between ethnicity and cellular proportions. Regression coefficients and p-values are reported as percentage change in child anthropometric outcome for 10% increase in percent methylation.

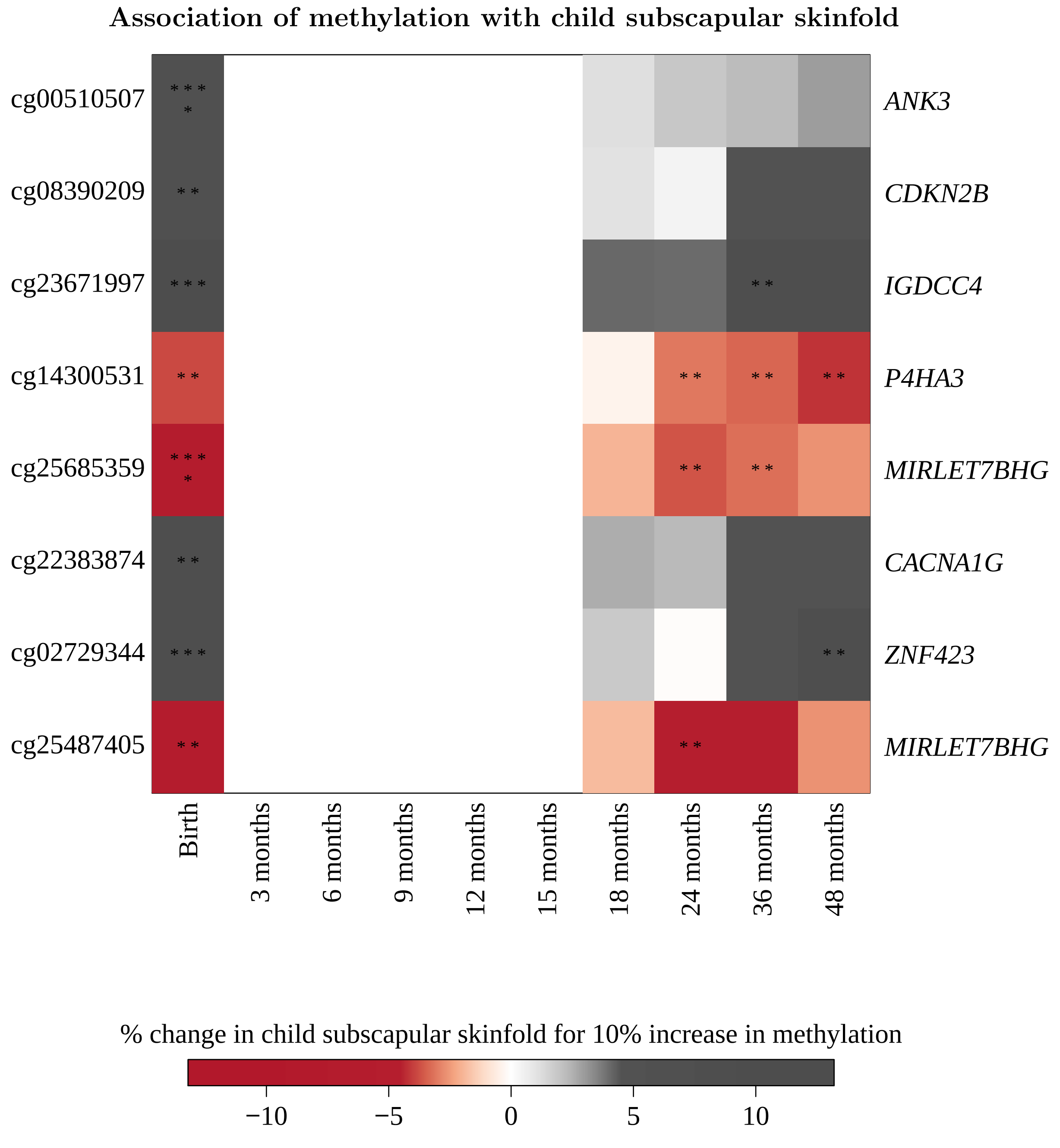

Supplementary Figure F2: **Influence of methylome at birth on triceps skinfold in early childhood.** Associations of child triceps skinfold at different time points with DNA methylation at birth. Color in heatmap represents regression coefficients for associations between child anthropometric outcome and methylation. Each row represents a CpG and each column represents a time point. With increasing magnitudes, color changes from white to red (for negative coefficients) or from white to grey (for positive coefficients). Asterisks within each square represent p-values for associations between child anthropometric outcome and methylation ($P < 5 \times 10^{-8}$ is represented with 8 asterisks, $5 \times 10^{-8} \leq P < 5 \times 10^{-7}$ is represented with 7 asterisks, $\cdots$, $5 \times 10^{-3} \leq P < 5 \times 10^{-2}$ is represented with 2 asterisks, $P \geq 5 \times 10^{-2}$ is represented with a blank square). Analysis was done by linear regression of log-transformed child anthropometric outcome at each time point against methylation at each CpG site, adjusted for child sex, gestational age, ethnicity, cellular proportions and interactions between ethnicity and cellular proportions. Regression coefficients and p-values are reported as percentage change in child anthropometric outcome for 10% increase in percent methylation.

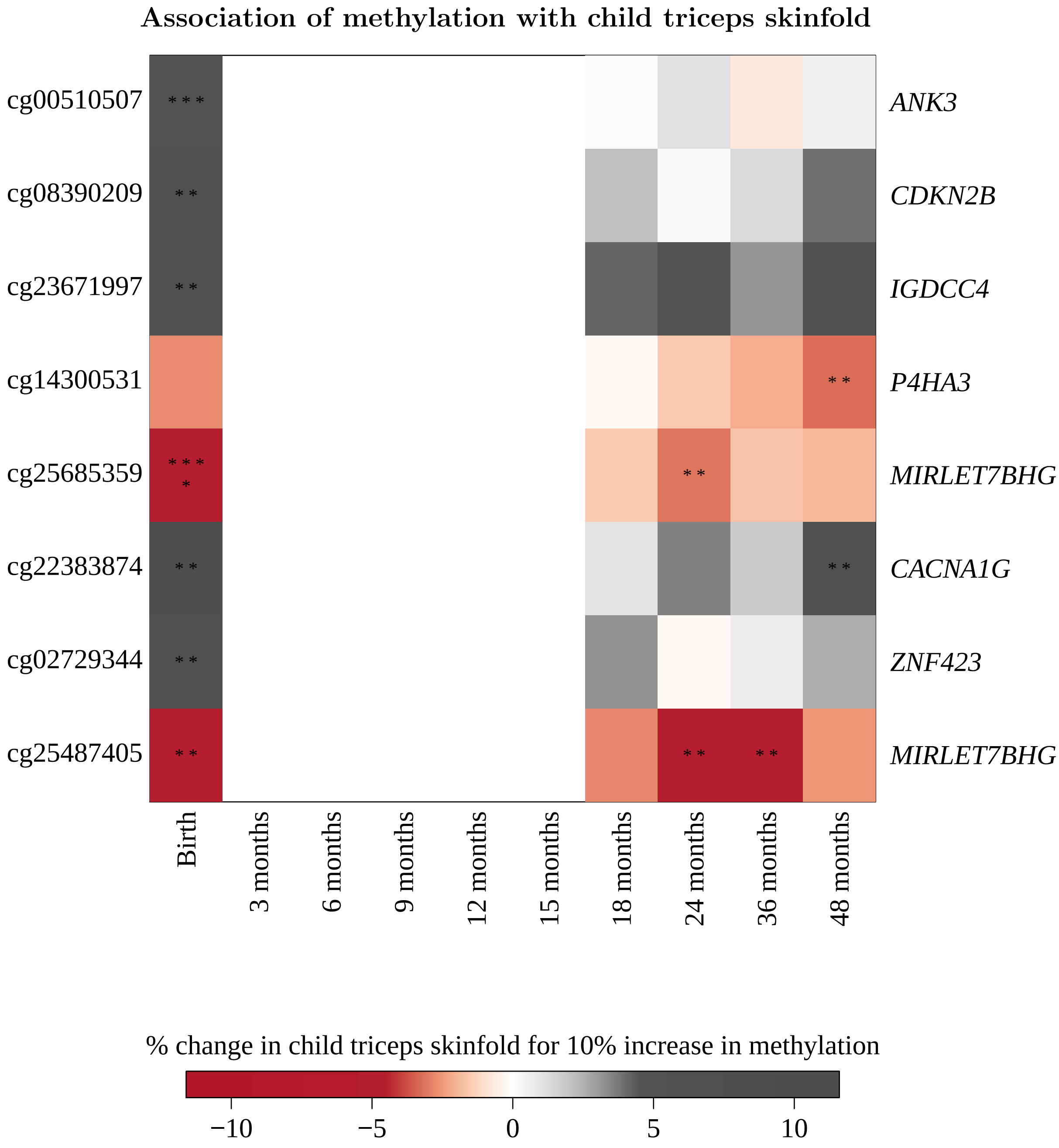

Supplementary Figure F3: **Influence of methylome at birth on subscapular:triceps ratio (STR) in early childhood.** Associations of child STR at different time points with DNA methylation at birth. Color in heatmap represents regression coefficients for associations between child anthropometric outcome and methylation. Each row represents a CpG and each column represents a time point. With increasing magnitudes, color changes from white to red (for negative coefficients) or from white to grey (for positive coefficients). Asterisks within each square represent p-values for associations between child anthropometric outcome and methylation ($P < 5 \times 10^{-8}$ is represented with 8 asterisks, $5 \times 10^{-8} \leq P < 5 \times 10^{-7}$ is represented with 7 asterisks, $\cdots$, $5 \times 10^{-3} \leq P < 5 \times 10^{-2}$ is represented with 2 asterisks, $P \geq 5 \times 10^{-2}$ is represented with a blank square). Analysis was done by linear regression of log-transformed child anthropometric outcome at each time point against methylation at each CpG site, adjusted for child sex, gestational age, ethnicity, cellular proportions and interactions between ethnicity and cellular proportions. Regression coefficients and p-values are reported as percentage change in child anthropometric outcome for 10% increase in percent methylation.

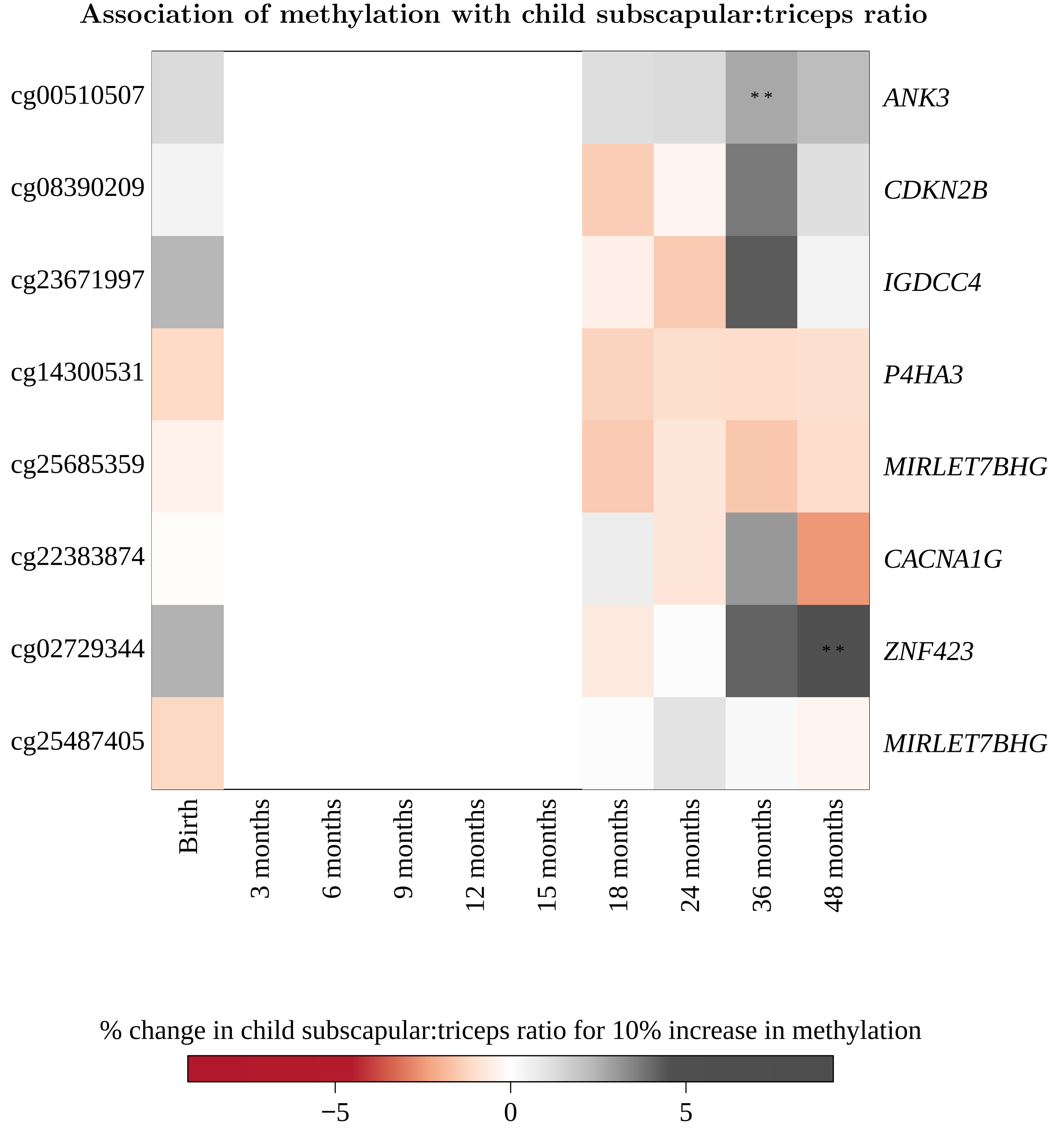

Supplementary Figure F4: **Influence of methylome at birth on length/height in early childhood.** Associations of child length/height at different time points with DNA methylation at birth. Color in heatmap represents regression coefficients for associations between child anthropometric outcome and methylation. Each row represents a CpG and each column represents a time point. With increasing magnitudes, color changes from white to red (for negative coefficients) or from white to grey (for positive coefficients). Asterisks within each square represent p-values for associations between child anthropometric outcome and methylation ($P < 5 \times 10^{-8}$ is represented with 8 asterisks, $5 \times 10^{-8} \leq P < 5 \times 10^{-7}$ is represented with 7 asterisks, $\cdots$, $5 \times 10^{-3} \leq P < 5 \times 10^{-2}$ is represented with 2 asterisks, $P \geq 5 \times 10^{-2}$ is represented with a blank square). Analysis was done by linear regression of log-transformed child anthropometric outcome at each time point against methylation at each CpG site, adjusted for child sex, gestational age, ethnicity, cellular proportions and interactions between ethnicity and cellular proportions. Regression coefficients and p-values are reported as percentage change in child anthropometric outcome for 10% increase in percent methylation.

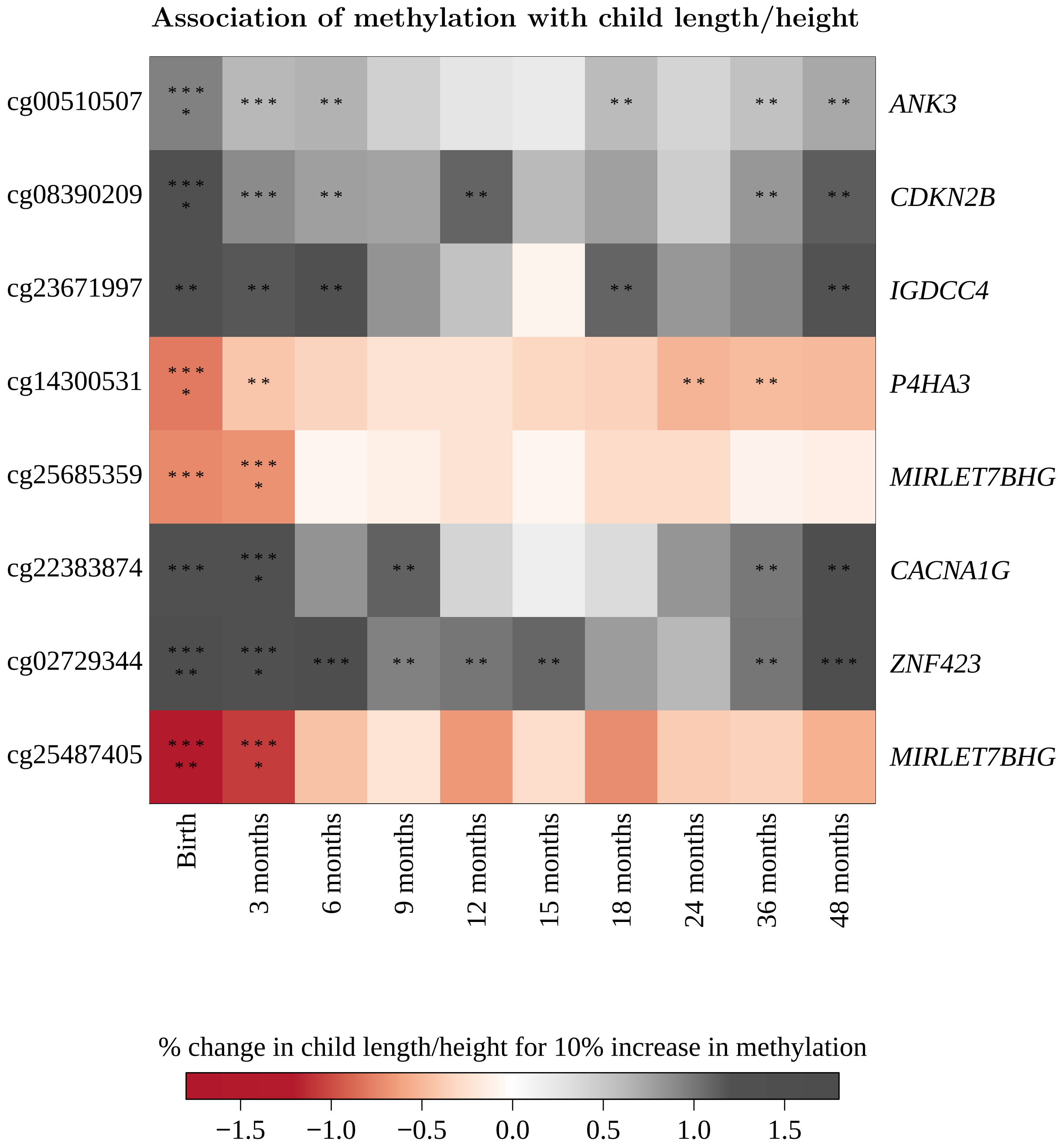

Supplement: Additional file 1: — Supplementary tables and figures. (PDF 2610 kb) [file 12916_2017_800_MOESM1_ESM.pdf]
