# Supplementary material for: Developmental pathways to adiposity begin before birth and are influenced by genotype, prenatal environment and epigenome
Source: BMC Med. 2017 Mar 7;15:50. doi: 10.1186/s12916-017-0800-1 (PMC5340003; doi:10.1186/s12916-017-0800-1)
Supplement: Additional file 3: — Information on using methylation data in Additional file 2. (PDF 42 kb) [file 12916_2017_800_MOESM3_ESM.pdf]

Developmental pathways to adiposity begin before birth and are influenced by genotype, prenatal environment and epigenome

Information on methylation file

- Compressed filename: GUSTO_Infant_450K_987samples_174211cpg.tar.gz (md5sum eaa9b17b9dd13f9bd594bdb406c6262d)
- To uncompress: "tar -zxvf GUSTO_Infant_450K_987samples_174211cpg.tar.gz".
- Uncompressed filename: v11b_data_GUSTO_450k_987samples-meth-blinded.pheno (md5sum: 442cfd5b4fa2ac6c56e710da50021979)
- Tab-delimited file contains methylation values for 987 samples, 174,211 CpGs.
- Missing value is coded as -9.
- Each row is a CpG, each column is a sample, first column gives CpG name.
